# Materials Quest for Advanced Interconnect Metallization in Integrated Circuits

**DOI:** 10.1002/advs.202207321

**Published:** 2023-06-15

**Authors:** Jun Hwan Moon, Eunjin Jeong, Seunghyun Kim, Taesoon Kim, Eunsoo Oh, Keun Lee, Hauk Han, Young Keun Kim

**Affiliations:** ^1^ Department of Materials Science and Engineering Korea University Seoul 02841 Republic of Korea; ^2^ Semiconductor R&D center Samsung Electronics Co., Ltd. Gyeonggi‐do 18448 Republic of Korea

**Keywords:** electrical resistivity, electromigration, integrated circuit, interconnect, metallization, semiconductor, size effect

## Abstract

Integrated circuits (ICs) are challenged to deliver historically anticipated performance improvements while increasing the cost and complexity of the technology with each generation. Front‐end‐of‐line (FEOL) processes have provided various solutions to this predicament, whereas the back‐end‐of‐line (BEOL) processes have taken a step back. With continuous IC scaling, the speed of the entire chip has reached a point where its performance is determined by the performance of the interconnect that bridges billions of transistors and other devices. Consequently, the demand for advanced interconnect metallization rises again, and various aspects must be considered. This review explores the quest for new materials for successfully routing nanoscale interconnects. The challenges in the interconnect structures as physical dimensions shrink are first explored. Then, various problem‐solving options are considered based on the properties of materials. New materials are also introduced for barriers, such as 2D materials, self‐assembled molecular layers, high‐entropy alloys, and conductors, such as Co and Ru, intermetallic compounds, and MAX phases. The comprehensive discussion of each material includes state‐of‐the‐art studies ranging from the characteristics of materials by theoretical calculation to process applications to the current interconnect structures. This review intends to provide a materials‐based implementation strategy to bridge the gap between academia and industry.

## Graphical abstract

This article presents a comprehensive review of the challenges faced by the back‐end‐of‐line in catching up with the rapidly shrinking front‐end‐of‐line. It systematically summarizes the considerations for rational design and reviews promising materials, from theoretical calculations to process applications. Finally, the article aims to provide an implementation strategy to bridge the gap between academia and industry.

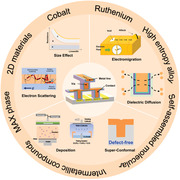

## 1 Introduction

Moore's prediction that the number of transistors in high‐density integrated circuits (ICs) doubles every two years has been a driving force in IC scaling until recently.^[^ ^1^, ^2^, ^3^, ^4^, ^5^ ^]^ However, with the latest entry into the fourth industrial revolution, the paradigm shift in semiconductor research is accelerating owing to the expansion of new application fields, such as big data, artificial intelligence, and the Internet of Things. These new applications demand low power consumption and high performance simultaneously. As IC scaling continues to progress, both the size of the transistor and the gap between the devices decrease, resulting in many problems, such as microcircuit pattern fabrication, quantum tunneling, increased wiring resistance, and heat generation.^[^ ^6^ ^]^ To solve these problems, engineers have continuously improved the degree of integration while overcoming the limitations of miniaturization technologies by using extreme ultraviolet light sources, heterogeneous integration of 3D structures, and stacked structures.^[^ ^7^, ^8^ ^]^ Nevertheless, as the physical area of a semiconductor device reduces to the nanoscale, the device's performance becomes significantly dependent on the exacerbation of electrical resistance rather than the processing speed of the transistor.^[^ ^9^, ^10^, ^11^ ^]^

The conductor line resistance (*R*) determines the level of electron transportation in interconnects, along with the parasitic capacitance (*C*) between metal lines separated by a dielectric medium. The *RC* product determines the time delay, which should be sufficiently small to transmit signals accurately. A helpful way to reduce the *RC* delay is to insert a low dielectric constant (*k*) material between the metal lines. Although initially, polymer‐based materials with very low dielectric constants were tested, they were incompatible with IC chip processing.^[^ ^12^, ^13^, ^14^, ^15^ ^]^ To address this, silica (SiO_2_, *k ≈* 3.9–4.2) has been replaced with materials containing C and H. Therefore, fluorine‐doped Si oxide (F‐SiO_2_, *k ≈* 3.0–4.0), nanoporous SiO_2_ (*k ≈* 1.3–2.5), organosilicate (SiCOH, *k ≈* 2.5–3.0), and porous organosilicate (p‐SiCOH, *k ≈* 2.4) were investigated.^[^ ^16^, ^17^, ^18^, ^19^ ^]^ Especially, SiCOH displays several benefits, which include a low dielectric constant, hydrophobic property, excellent thermal stability, good bonding with metal layers, and durability against cracking and delamination.^[^ ^20^, ^21^ ^]^ Materials containing N, such as SiON (*k ≈* 4.3–5.0) or SiOCN (*k ≈* 4.5–5.3), are used as capping layers for Cu lines or spacer dielectric materials.^[^ ^22^, ^23^ ^]^ Additional reduction in capacitance in the interconnect structure can be achieved by forming an air‐gap (*k ≈* 1.0). Studies are underway to explore the suitability of SiN, SiCN, and p‐SiCOH materials in creating an air‐gap by considering their mechanical strength, low leakage, excellent reliability, and robustness for integrated manufacturing.^[^ ^24^ ^]^ In addition to above mentioned Si‐based low‐k materials, the studies on organic or organic‐inorganic hybrid porous materials, such as 2D covalent organic framework (*k ≈* 1.2–1.6) and metal‐organic framework (*k ≈* 1.25–2.0), are gaining attention.^[^ ^25^, ^26^, ^27^, ^28^ ^]^ However, further improvements in manufacturing methods and mechanical aspects are necessary to use these materials in the actual process. Despite this, the interconnect capacitance has remained stable from one node to another in recent years.^[^ ^29^ ^]^ The *RC* delay of the circuit, which determines the performance quality of the IC, is primarily relevant to the electrical resistivity (*ρ*) of the material.^[^ ^30^ ^]^ In other words, using a material with low resistivity is crucial for reliably increasing the speed of the device.

**Figure 1:**
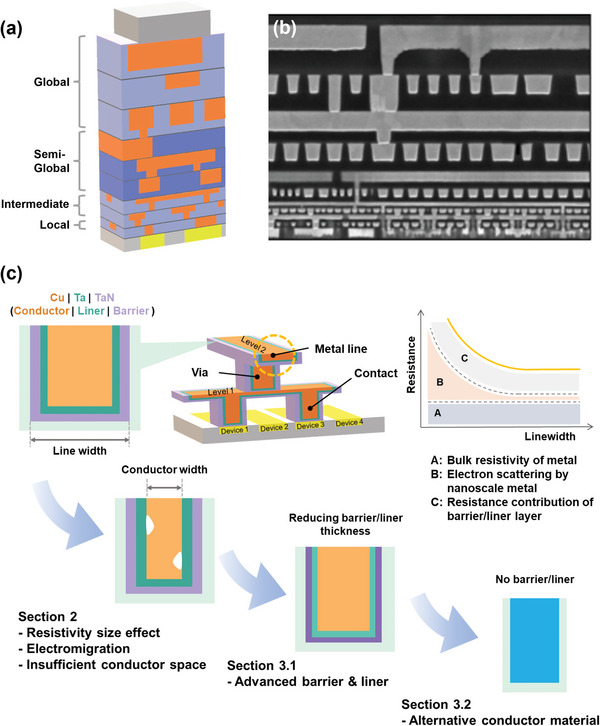
a) Schematic of the hierarchical interconnect structures of ICs. b) Cross‐sectional scanning electron microscopy image of the IC chip from Intel's 14‐nm process. Reproduced with permission.^[^ ^33^ ^]^ Copyright 2014, Institute of Electrical and Electronics Engineers. c) Schematic of the first two levels of the interconnect and multilayered structures (top left). Line resistance contribution graph according to scaling (top right). Classification of the interconnect research and framework of this article (bottom).

Over the past few decades, one of the most revolutionary developments in the back‐end‐of‐line (BEOL) processes of ICs has been the introduction of Cu metal to replace Al and low‐*k* materials to reduce the *RC* delay.^[^ ^31^, ^32^ ^]^ The BEOL represents the interconnection of the top part of a chip with complex wiring that distributes signals, provides power and ground, and transmits electrical signals from one transistor to another. Various metal layers, such as local, intermediate, and global interconnections, offer intricately embroidered electron paths (**Figure** **1**a,b).^[^ ^33^ ^]^ Since IBM first used Cu as an interconnect material in 1997, Cu has been used as a metal material until now due to its lower resistivity than Al, better electromigration (EM) durability, and suitability for dual‐damascene processing.^[^ ^34^, ^35^ ^]^ For instance, a report suggested that using Cu as a core material in interconnect metallization could decrease wiring resistance by up to 45%. Concerning EM, Cu showed a 100‐fold increase in the time required for 50% of the devices to fail at a high temperature (295 °C) and a stress current of 2 500 000 A cm^−2^ compared to the Ti/Al(Cu)/Ti line. The terrestrial abundance of Cu and its superior electrical conductivity have maintained the same timeline for transistor scaling. However, the additional problem followed as the BEOL dimension decreased rapidly, incurring a shrinkage of pitch and the cross‐sectional area of the interconnect to overcome the area scaling in the front‐end. In the case of a 5 nm node, the interconnect resistance affects ≈50% of the total RC delay.^[^ ^36^, ^37^ ^]^ These problems accelerate as the chip is miniaturized, particularly for an initial metal wiring connected to metal zero (M0) among multiple interconnect stack structures for external connection with the contact area of a metal material first contacting a semiconductor device the most serious. Therefore, it is clear that improving metal interconnections is one of the main targets of modern research on electronics, given the electronics industry's significant economic and social impact.^[^ ^38^ ^]^

To keep pace with the area reduction achieved in the front‐end, the interconnect line width of the lowest layer of semiconductor devices becomes tighter down to 20 nm. This reduction in dimensions leads to significant multifaceted problems (Figure 1c). The first problem is the resistivity size effect. The resistivity of Cu interconnects rapidly increases as the interconnect linewidth decreases below the electron mean free path (EMFP) in bulk Cu.^[^ ^39^, ^40^ ^]^ For instance, when the linewidth of a Cu interconnect is reduced to 10 nm, electron scattering at the surfaces and grain boundaries (GBs) causes about a ten‐fold increase in line resistivity value compared to bulk resistivity value.^[^ ^41^, ^42^ ^]^ Increased resistivity increases both power consumption and speed delay, thereby limiting IC device performance and hampering scaling. Second, as the current density and device operating temperature increase due to the increased electron scattering by the reduced area, EM, or stress voids severely damage the reliability of the interconnect material. The gradual transfer of momentum induces the movement of metal atoms, creating unintended electrical connections that cause circuit malfunctions. Finally, reducing the critical dimension (CD) of the interconnect significantly increases the volume ratio of the additional layers required, causing process difficulties and accelerating the increase in resistance. The minimum thickness required for a proper barrier/liner function is 3–4 nm. The additional layers complicate the formation of high‐aspect‐ratio vias or contacts in the trench structure. Moreover, when the trench width is 20 nm and the aspect ratio is 2, the cross‐sectional area occupied by the 3‐nm‐thick Ta/TaN structure is >30%.^[^ ^43^ ^]^ This multilayer structure results in a more than three‐fold increase in the 10‐nm half‐pitch Cu line resistance.^[^ ^41^, ^42^, ^44^ ^]^

These motivations once again call the quest for alternative materials for interconnect metallization. Routing congestion and dramatic *RC* delays have become significant bottlenecks for further interconnection expansion, which creates the need to introduce new materials and integration plans for BEOL. Since the scaling issues of interconnects depend on the properties of nanoscale materials, innovative interconnection property improvement can be possible through material changes, and its compatibility with the complementary‐metal‐oxide‐silicon (CMOS) process is also essential. The following sections discuss the materials science aspects of the interconnect materials.

**Figure 2:**
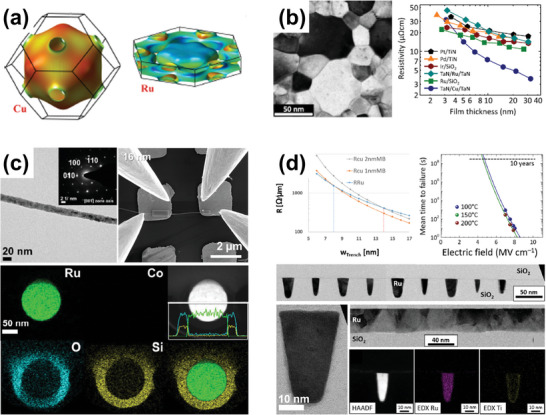
Examples of versatile research methods for interconnect materials. a) The Fermi surfaces of Cu and Ru, as obtained from first‐principles calculations. Reproduced with permission.^[^ ^39^ ^]^ Copyright 2016, American Institute of Physics publishing. b) Transmission electron microscopy (TEM) image of the 30‐nm‐thick 2D films of Ru/SiO_2_ deposited by PVD and the resistivity changes of Pt‐group metals as a function of film thickness. Reproduced with permission.^[^ ^45^ ^]^ Copyright 2017, American Institute of Physics publishing. c) The electrodeposited Co nanowire with a diameter of 16 nm and its electrical resistivity measurement (upper). Reproduced with permission.^[^ ^53^ ^]^ Copyright 2020, Elsevier. Elemental mapping images and line profiles of cross‐sectional Ru‐SiO_2_ core‐shell nanowire (lower). The electrodeposited Ru nanowire did not inter‐diffuse into SiO_2_ after annealing at 450 °C for 3 h. Reproduced with permission.^[^ ^54^ ^]^ Copyright 2022, Elsevier. d) Line resistance, time‐dependent dielectric breakdown, and cross‐sectional TEM image of the Ru interconnect fabricated by ALD in the trench structure. Reproduced with permission.^[^ ^63^ ^]^ Copyright 2016, American Chemical Society.

The areas related to interconnect research are divided according to several situations as follows (**Figure** **2**). 1) To deal with the arousing problems due to the continuous scaling of interconnects, new materials are considered through classical calculations and theoretical modeling.^[^ ^39^, ^44^, ^45^, ^46^ ^]^ This consideration includes theories depending on the material types and crystal structures, nanoscale electron scattering mechanism, grain boundary (GB) modeling, and particle behavior in electric fields. 2) Based on the abovementioned theories and calculations, promising next‐generation materials are compared by conducting thin‐film experiments using various deposition methods. Thin films deposited by physical vapor deposition (PVD), chemical vapor deposition (CVD), electrodeposition (ED), and atomic layer deposition (ALD) allow practical observations on the nanoscale.^[^ ^45^, ^47^, ^48^, ^49^, ^50^, ^51^, ^52^ ^]^ In this process, some studies insist that there are some breakdown and expectation deflections in classical theories. 3) Therefore, the selected materials by thin‐film experiments go through the advanced process to verify their narrow line resistance (one‐dimensional or geometry) and scaling potential.^[^ ^53^, ^54^, ^55^, ^56^, ^57^ ^]^ It analyzes the properties required for the wiring structure by simulating the metal line structure or fabricating a 1D linear metal using the etching and bottom‐up fill process. 4) Consequently, the metallization module development for the trench or via structure application proceeded.^[^ ^58^, ^59^, ^60^, ^61^, ^62^, ^63^ ^]^ This process performs a comparative analysis of the current metallization structure and the line or via resistance, EM, and time‐dependent dielectric breakdown (TDDB). Moreover, this stage also includes the actual process and precursor development to adapt the promising materials to real devices.

This article focuses on the material quest and comprehensively reviews the interconnect development trends that seek to keep up with aggressive downscaling. Figure 1c summarizes what this review article intends to address. We begin by discussing the fundamental physical mechanisms of the resistivity size effect and the reliability problems nanoscale interconnects face. Then, since the transition of central materials necessitates major process procedures and design changes, we discuss the continuation of next‐generation barriers and liners for Cu interconnect scaling by reducing the number of layers or their thickness. Subsequently, we consider a solution for the two problems based on the inherent properties of the materials, and research results related to promising conductor materials are broadly presented based on the two proxies. Each discussion of the materials ranges from theoretical studies based on first‐principles calculations to the availability of actual interconnect process applications. Finally, we summarize the challenges, opportunities, and new directions for future development.

## 2 Interconnect Materials and Critical Issues

### 2.1 Electron Scattering by Surface and Grain Boundary

**Figure 3:**
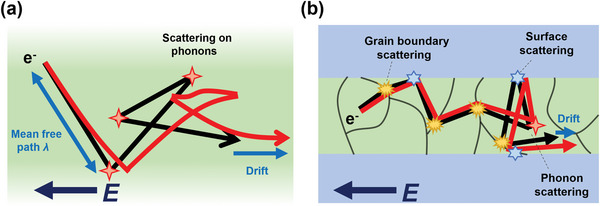
Scattering mechanism on a) bulk and b) line or thin film at nanoscale. Additional electron scattering at the surfaces and grain boundaries interferes with electron movement and increases resistivity.

This section discusses the resistivity size effect faced by metal lines (or conductors) and effective electron transport paths against interconnect scaling. Recently, new quantum mechanical models have been developed to overcome the limitations of classical models, such as the one based on the Boltzmann transport equation (BTE) in narrow metal lines.^[^ ^64^ ^]^ To briefly explain, the quantum theory of size effect uses quantum mechanics to describe electron motion and completely ignores the existence of GBs. This theory aims to build a formalism based entirely on quantum mechanics that can predict increased resistivity due to rough surfaces and electron collisions. The quantum description of GB scattering begins with Green's function built to solve the Schrödinger equation in a 1D potential representing uniformly spaced grains. However, most discussions use classical and semiclassical models based on the BTE because they can offer better insights into how the surfaces and GBs affect the changes in electrical resistivity values. The widely used classical model fails to quantitatively predict the increase in resistivity of narrow metal lines (thickness < 10 nm) compared to the experimental values.^[^ ^65^, ^66^ ^]^ Nevertheless, the classical model directly transforms a material property into an understandable concept that can handle the cause of increasing resistivity. In the bulk state, electron scattering by phonons primarily determines the bulk EMFP of each material (**Figure** **3**). However, as the dimension of the material decreases, electron scattering becomes more pronounced at the surfaces and GBs.^[^ ^41^, ^64^, ^67^, ^68^ ^]^

Therefore, according to Matthiessen's rule, the nanoscale resistivity is the sum of contributions to the bulk resistivity of each material from electron scattering by the external surface, predicted by Fuchs and Sondheimer (FS model)^[^ ^69^ ^]^ and by the GB, predicted by Mayadas and Shatzkes (MS model)^[^ ^70^, ^71^ ^]^ (Equation (1)):

(1) $$\rho_{\mathrm{total}}=\rho_{0}\;+\;\Delta \rho_{\mathrm{FS}}+\Delta \rho_{\mathrm{MS}}$$

(2) $$\rho_{\mathrm{FS}}=\rho_{0}\;{\left[1-\left(\frac{3\lambda }{2d}\right)\left(1-p\right)\int_{1}^{\infty }\left(\frac{1}{t^{3}}-\frac{1}{t^{5}}\right)\frac{1-\exp\left(-kt\right)}{1-p\exp\left(-kt\right)}dt\right]}^{-1}\;\mathrm{where}\;k=\frac{d}{\lambda }$$

(3) $$\rho_{\mathrm{MS}}=\rho_{0}\;{\left[1-\frac{3}{2}\alpha +3\alpha^{2}-3\alpha^{3}\ln\left(\frac{1}{\alpha }\right)\right]}^{-1}\;\mathrm{where}\;\alpha =\left(\frac{\lambda }{D}\right)\left(\frac{R}{1-R}\right)$$

Here *ρ*_0_ is the bulk resistivity that quantifies electron relaxation due to phonon scattering, *λ* is the EMFP of the metal, *d* is the thickness of the metal, and *D* is the average grain size. By applying the first‐level approximation to Equation (3), the resistivity value of a square wire of length *d* on one side can be simplified as follows:^[^ ^49^ ^]^

(4) $$\rho_{\mathrm{total}}=\rho_{0}+\rho_{0}\lambda \frac{3\left(1-p\right)}{4d}+\rho_{0}\lambda \frac{3R}{2D\left(1-R\right)}$$

In the above equation, the nanoscale resistivity increase is primarily due to the contribution of three variables: the surface scattering parameter (*p*), the GB scattering parameter (*R*), and the electronic structure of a specific metal (*ρ*_0_ × *λ*).

In the classical model, the electrons are assumed to be particles; therefore, electron scattering at the interface between the conductor and the liner layer depends on the random variable surface scattering parameter (*p*). This phenomenological secularity parameter *p* represents the momentum parallel to the drift direction of electrons at the conductor interface. The diffuse scattering parameter (1 − *p*) represents the momentum of lost electrons, contributing to the increase in resistivity. Therefore, specular scattering (*p* = 1) of electrons at the interface does not contribute to increased electrical resistivity, which is desirable for designing high‐conductivity interconnects. The phenomenological approach to surface scattering involves a change in the Fermi surface for electrons in the region near the surface, affected by atomic‐level roughness and atoms on the surface, especially atoms of other species.^[^ ^72^ ^]^ The electrons are considered waves if we interpret this phenomenon using a semiclassical model. When a plane wave of incident electrons is focused on the interface, it generates an array of circular waves that constructively interfere with forming a reflected plane wave. Thus, specular scattering of the electrons occurs.^[^ ^46^ ^]^ This phenomenon is called an electron mirror, and several requirements exist to satisfy the generation condition. First, the interface surrounding the conductor must be atomically smooth. In de Broglie's material wave theory, because electrons have wavelengths, atomic‐scale roughness causes destructive interference of reflected waves, contributing to increased resistivity.^[^ ^65^, ^73^, ^74^, ^75^ ^]^ Adsorbents on a metal surface change the surface roughness, and the generated local potential increases the scattering of conduction electrons.^[^ ^76^, ^77^, ^78^, ^79^, ^80^ ^]^ In general, conductor metal is surrounded by a barrier/liner or capping layer and chemically bonds to form interfaces. High conductivity could be achieved when the conductor metal has high electronegativity due to undisturbed flat potential drop where less occurrence of surface charge transfer can have a smoother surface. These metals include Mo, Ru, Rh, W, and Ir.

Next, the *p*‐values of the metallic conductors decrease when designed to have a low density‐of‐state (DOS) or superposition of negligible wavefunctions at the liner interface. For example, perfectly inelastic electron scattering occurs at the interface of Cu and metals with high DOS (such as Ta) because the complex Fermi surfaces of high‐DOS metals disturb the smooth surface potential of Cu, thus facilitating electronic coupling between them.^[^ ^81^ ^]^ In contrast, the DOS of Al and Pd barrier atoms correspond to that of Cu atoms with a perfectly flat surface, inducing more specular scattering at the interface and lower resistance than that of the Cu film.^[^ ^82^ ^]^ However, the locally scattered electrons in the conductive liner return to the metal conductor with a random momentum, resulting in diffuse scattering. In contrast, the insulating liner suppresses this scattering mechanism, promoting specular scattering.^[^ ^83^, ^84^ ^]^ Therefore, there is a need for an insulating liner that does not interfere with the flat metal surface or cause localized DOS changes at the Fermi level. It can be achieved by creating an epitaxial liner interface with a specific conductor metal or using 2D materials with weak van der Waals bonding as the liner.^[^ ^43^, ^85^, ^86^, ^87^ ^]^ Specifically, weak interaction between the Cu surface and graphene or h‐BN layers prevents surface oxidation of Cu and preserves the Shockley surface states, thereby reducing interface scattering.^[^ ^88^ ^]^ The Cu/MoS interface has a lower localized DOS than the Cu/amorphous SiO_2_ interface, thus improving the electrical performance of Cu by up to 40% due to improved specular surface scattering.^[^ ^89^ ^]^

In the FS model, surface scattering is the interference of waves at the interface between the central conductor surface and the layer surrounding the central conductor. For perfect reflection, the electron wave must be an electron mirror, which creates constructive interference at the conductor interface. An electron mirror can be obtained by designing an atomic‐level smoothness, choosing materials with negligible wavefunctions overlap or liner/barrier DOS at the Fermi level, and an undisturbed flat potential drop via barrier and lattice match.

**Figure 4:**
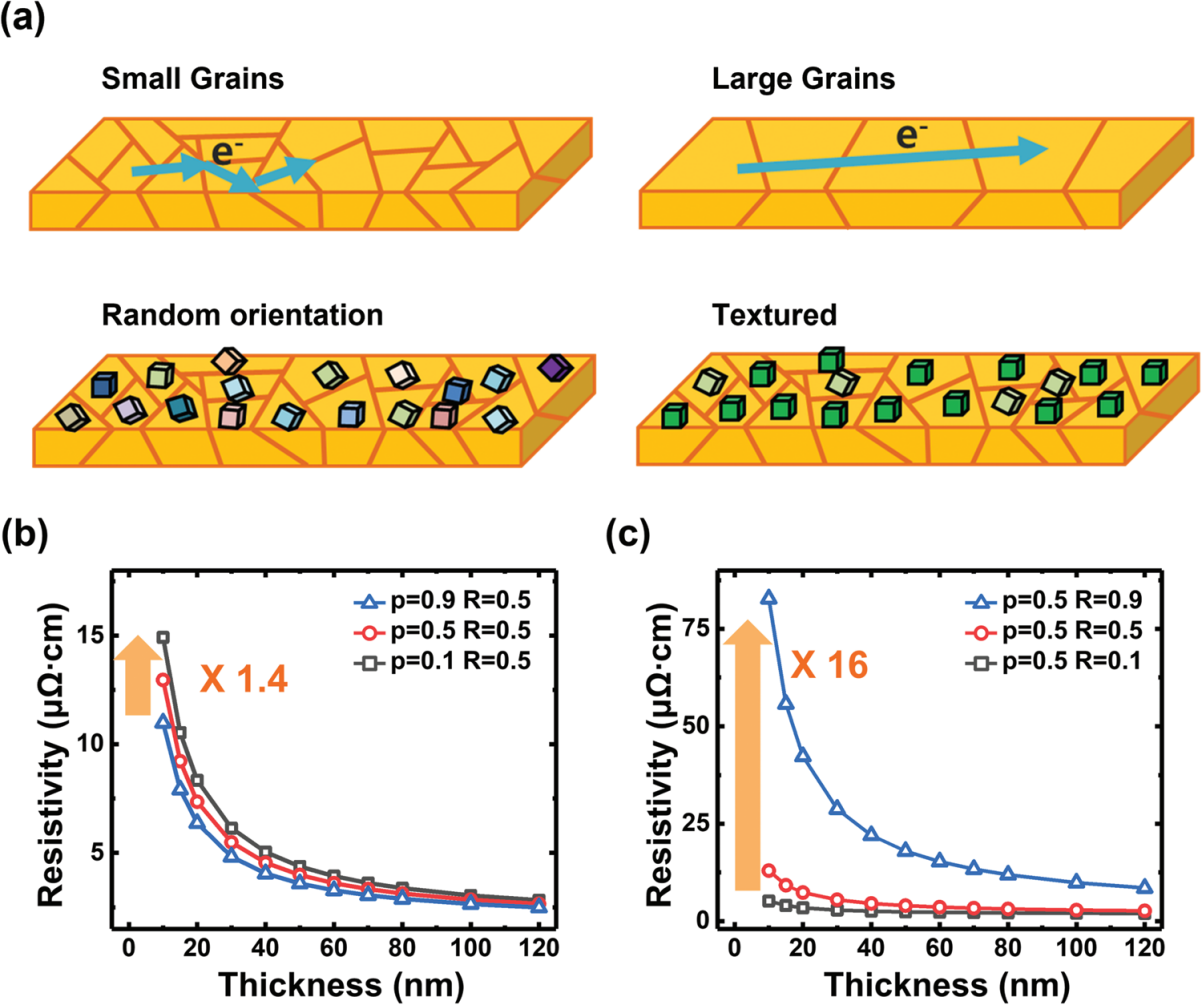
a) Schematic of electron transmission according to grain engineering. Electron scattering depends on grain size as well as orientation. b,c) Changes in resistivity of Cu (*λ*_Cu_ = 39.9 nm and *ρ*_0, bulk_ = 1.68 µΩ cm) film according to changes in the values of two types of electron scattering parameters in the classical FS‐MS model. Resistivity changes according to the b) surface and c) GB scattering parameters. The thin film thickness and grain size were assumed to be identical in both graphs. Note that these are the classical model results for the 2D thin film with many assumptions, and both scatterings are important factors in the actual interconnect structure.

On the other hand, the MS model accounts for the nanoscale resistivity increase in terms of the phenomenological GB reflection parameter *R* and the grain size *D*. This model extends the Boltzmann transport theory and predicts the resistivity contribution of the reflection and transmission of conduction electrons at polycrystalline metal GBs. The GBs are assumed to be parallel or perpendicular to all current flow directions, and only the parallel GB incident electrons are specular.^[^ ^71^ ^]^ Accordingly, the GB reflection coefficient has a value between that where the resistivity increases to infinity because all electrons are reflected (*R* = 1) and that where there is no additional increase in resistivity because all electrons are transmitted (*R* = 0). The average *R*‐value is quantified experimentally based on the correlation between the resistivity and the grain size distribution. Indeed, materials engineering designs are also required to reduce GB scattering (**Figure** **4**). For example, when diffusive scattering occurs on the surface of 10‐nm‐thick films, the resistivity increases to ≈1.4 times more than that of the specular‐scattering‐dominant thin films. In contrast, the change in GB scattering results in an approximately 16‐fold increase in resistivity (Figure 4b,c). Increasing the grain size is one way to decrease the grain scattering frequency. If a narrow wire has tiny grains, the GB density increases accordingly, leading to considerable reflection at the GBs. However, when the grain size is larger than the bulk EMFP of the metal, the scattering at the GB is negligible compared to electron‐phonon scattering. In addition, if the grain size is ten times larger than the conductor thickness, the main contribution to resistivity is scattering from the conductor surface.

Instead of controlling grain sizes, a small *R* leads to an insignificant MS model contribution to resistivity. Unlike the scattering inside the grain, the GB geometry that loses the periodicity of the atomic structure affects the *R*‐value.^[^ ^90^, ^91^, ^92^ ^]^ When electrons drift from one crystal to another across the boundary, the electron scattering occurs by the potential wall resulting from the locally changed DOS and electron density compared with the grain interior.^[^ ^93^ ^]^ Although a high potential wall increases *R* and thereby aggravates the resistance, if the potential is small, the electron will transmit rather than be reflected at the GB, resulting in low resistivity. In thermodynamics, the GB phase is a function of excess interfacial energy, excess volume, excess entropy, and interfacial stress. The misorientation between adjacent grains significantly affects the equilibrium GB structure.^[^ ^94^ ^]^ Several stable and metastable states exist for different GB orientations, and particular GBs in metals have only recently been observed to have different phases.^[^ ^95^ ^]^ A conductive scanning probe method makes it possible to measure the specific resistivity of individual GB.^[^ ^96^, ^97^, ^98^ ^]^ GB resistivity is closely related to the coincident‐site lattice type, which indicates a specific periodic atomic structure by tilting it to a particular angle. The GB resistivity increases with the excess volume of the coincident‐site lattice.^[^ ^99^ ^]^ For example, **Table** **1** lists the specific resistivities and GB reflection parameters for Ru, Co, and Cu coherent boundaries in a face‐centered cubic (*fcc*) structure.^[^ ^100^, ^101^, ^102^ ^]^ Moreover, the size of the potential wall is determined by its chemical bonding and GB characteristics, such as curvature, gradient, phase, and defects. The GB curvature increases the boundary resistance to 80% due to the deviation from the gradient condition.^[^ ^99^ ^]^ Suggestions to induce charge compensation, such as doping with foreign elements, have been proposed to lower the GB potential wall.^[^ ^93^ ^]^

**Table 1:** Calculated GB‐specific resistivities (×10^−12^ Ω cm^2^) and reflection coefficients of promising interconnect metals at several coincident‐site lattices

	Σ3		Σ5		Σ9		Σ11	
Metal	*γ*_GB_	*R*	*γ*_GB_	*R*	*γ*_GB_	*R*	*γ*_GB_	*R*
Ru^[^ ^88^ ^]^	7.67	0.26	9.73	0.46	9.68	0.50	8.66	0.43
Co^[^ ^89^ ^]^	9.70	0.20	11.9	0.38	10.7	0.33	9.60	0.28
Cu^[^ ^90^ ^]^	0.22	0.02	1.32	0.13	1.80	0.14	0.64	0.07

In the MS model, the strength of scattering potential at the boundary is explained by the loss of atomic periodicity and the alteration of the Fermi velocity of electrons propagating in different directions. In detail, the electrons that collide at the boundary with a transport‐related Fermi energy *E*_f_ and momentum *p* will not be scattered if there is an empty state at the new GB.^[^ ^49^ ^]^ The GB crossing corresponds to the rotation of the Fermi surface, which is related to the crystal rotation. Therefore, to achieve non‐scattering transmission, the low‐energy boundary should be formed by constructing a round‐shaped Fermi surface, highly symmetric boundaries, or textured grains by matching the Fermi surface between two different grains.

### 2.2 Electromigration

Electromigration (EM), the movement of atoms based on electron flow in a potential gradient, is one of the primary considerations for selecting conductor materials as interconnect scaling continues, along with the resistance.^[^ ^103^, ^104^, ^105^, ^106^ ^]^ Reducing the conductor thickness to a few nanometers reduces the cross‐sectional area; this increases the current density dramatically, which causes EM. The potential gradient inside the narrowed‐down metallization line increases the collision between the flowing electrons and metal atoms, resulting in a severe momentum transfer.^[^ ^107^ ^]^ The heat generated by the reinforced current density inside the material continuously decomposes and causes the atoms in the structure to migrate.^[^ ^108^, ^109^ ^]^ The migrated metal atoms create vacancies and deposits inside the wire.^[^ ^110^ ^]^ The continuous increase in vacancies eventually disconnects the circuits, resulting in open circuits, whereas the increase in deposits helps close the circuit connection, causing shorts. Gerardin discovered this phenomenon for the first time in 1861,^[^ ^111^ ^]^ and it was considered a severe reliability problem in an Al‐based IC in 1967.^[^ ^112^ ^]^ At that time, the width of the metal interconnects was still 10 µm; however, for now, the width is scaled down to only a few nanometers, increasing its importance.

**Figure 5:**
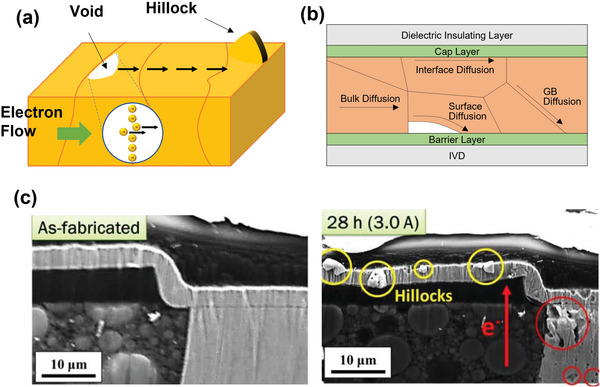
Failures caused by electromigration. a) Illustration of the depletion of atoms (voids) resulting in an open circuit and deposition of atoms (hillocks) causing a short. b) Various pathways whereby electromigration occurs in metal lines. c) The results of the stepwise electromigration experiment of the local Cu line/Cu tall pillar. Reproduced with permission.^[^ ^113^ ^]^ Copyright 2021, Institute of Electrical and Electronics Engineers.

Failure will not occur if uniform EM is maintained within the metallization line. Because the number of atoms reaching a given local volume along the metallization line is equal to that leaving the volume, no damage should be observed in the normal state except at the start and endpoints. However, the damage to the metallization line is caused by the divergence of atomic flux. The difference in the number of atoms that enter and exit at a given volume results in two types of inequality (**Figure** **5**).^[^ ^113^ ^]^ The slow decline of the connectivity or the failure of interconnects is due to atomic scarcity (void) and shorts caused by atomic deposition (hillock).^[^ ^114^ ^]^

In the late 1960s, Black developed a semi‐empirical model to estimate the mean‐time‐to‐failure (MTTF) of the wire by considering EM:^[^ ^112^ ^]^

(5) $$\mathrm{MTTF}\;=\;\frac{A}{J^{n}}\exp\left(\frac{E_{a}}{k_{B}T}\right)$$

where *A* is a pre‐exponential constant based on the geometry‐dependence of the interconnect, *J* is the current density, *n* is an integral constant (1 or 2), *E*_a_ is the activation energy, *k*_B_ is the Boltzmann constant, and *T* is the temperature in Kelvin. According to Black's equation, the temperature of the conductor is expressed in an exponential form, that is, it significantly impacts the MTTF of the interconnect. The interconnect temperature is primarily related to the chip environment temperature: the heat released from the current flow, adjacent current, or transistor, and the thermal conductivity of the surrounding material. Therefore, the current density should be decreased to maintain a stable state, even though the temperature of the interconnects in the given structure increases. Determining activation energy and current density index through experiments is crucial, as questions arise about the equation's validity as interconnect technology advances. The durability of interconnects is primarily affected by: the material of the metallization line and the shape of the conductor related to its dimensions; the crystallographic orientation of the metal grains; the layer deposition and annealing processes; passivation properties; the interface properties with other materials.^[^ ^113^, ^115^ ^]^ A physical approach based on the real interconnect structure diffusion should be applied to elucidate this phenomenon.

In an electric field, the conductor is affected by the direct electrostatic force (*F*_e_) generated in the same direction as the electric field, and the electron wind or ion wind force (*F*_w_), generated in the opposite direction as the electric field under the influence of momentum exchange with other charge carriers.^[^ ^116^, ^117^ ^]^ Therefore, the total driving force *F*_tot_ that the activated ion inside the electric field receives is

(6) $${\vec{F}}_{\mathrm{tot}}={\vec{F}}_{e}\;+\;{\vec{F}}_{w}=\;q\cdot \left(Z_{e}-Z_{w}\right)\cdot \vec{E}\;=\;q\cdot Z^{*}\cdot \vec{E}\;=\;q\cdot Z^{*}\cdot \rho \cdot \vec{J}$$

where *q* is the electric charge of the ions, *Z*_e_ and *Z*_w_ are the valences corresponding to the electrostatic and wind forces, respectively, Z* is the effective valence of the material, *J* is the current density of the conductor, and *ρ* is the resistivity of the material. Each material for a specific technology node requires a discrete calibration to manage any effects during manufacturing and operation.^[^ ^118^, ^119^ ^]^

The metal atoms migrate when some of the momenta of electrons moving inside the electric field are transferred to a neighboring activated metal ion. Generally, in a uniform crystal structure, in which the lattice of metal ions is arranged, there is minimal transfer of momentum between conduction electrons and metal ions; therefore, high activation energy is required to change the lattice site. The activation energy is primarily related to the cohesive energy of the conductor material. Metals with high melting points and cohesive energies have excellent ionization resistance to electron wind because of their strong atomic lattice bonds. However, the metal atoms are weakly bonded at the GB and material interface, where the crystal symmetry is disrupted, compared to that in the typical crystal lattice. Therefore, when the strength of the electric wind reaches a certain point, the atoms are readily disassembled and moved along the electric current direction.

Apart from the nanoscale electrical resistivity, the microstructure of the metal conductor also significantly affects the EM properties. In the previously presented models, the stress distribution was assumed to be independent of the shape, direction, and size variation of the grains. Unfortunately, because adjacent grains have different crystal orientations, individual grain in the same material exhibits varying behavior when applying a mechanical load.^[^ ^120^ ^]^ The diffusion caused by EM can be classified into GB, surface, interfacial, and bulk diffusion (Figure 5b). In the current interconnect material, for example, the Cu ion activation energy of the bulk (within the grains) is 2.1 eV. In contrast, the surface, material interface, and GB are 0.5–0.7, 0.8–1.25, and 1.2–1.25 eV, respectively.^[^ ^119^, ^121^, ^122^, ^123^, ^124^, ^125^ ^]^ The diffusion constant of Cu ions at the surface is three times the diffusion constant inside the grain and approximately twice the diffusion constant at the material interface. Therefore, to accurately predict reliability, the regions near the surface and material interfaces must be strictly considered for the diffusion of metal ions.

Grain engineering methods have been proposed to address these metal ions. One method involves preventing the movement of atoms by alloying Cu with metals like Al, P, Mn, and Sn.^[^ ^126^, ^127^ ^]^ For instance, *β*‐Sn particles formed between Cu GBs have high diffusion anisotropy. Cu movement increases when electrons flow from a direction where the angle between the *c*‐axis of *β*‐Sn particles and the electron flow direction is low to a high angle. In contrast, when electrons flow from a high angle to a low angle, there is almost no movement of Cu.^[^ ^127^ ^]^ Some low concentrations of Mn diffuse into the GBs of Cu, inhibiting Cu from diffusing along grain boundaries. Another method involves constructing a bamboo structure inside the conductor via chemical mechanical polishing (CMP). When the wire width is reduced below the average grain size of the wire material, theGBs become transverse and perpendicular to the long axis of the wire. Ironically, despite increasing the current density, the resistance to material movement in this structure increases. This is because it creates an ideal microstructure while minimizing the GBs not perpendicular to the average current density vector. Grain size control of conductors has been attracting attention at the continuous technology node. As explained in the previous section, the larger grain size leads to lower electrical resistivity and a lower grain boundary diffusion contribution to electron movement. A method for making fine Cu to have the ideal bamboo structure has not yet been developed. As an alternative to this, research is underway to identify alternative materials with excellent EM performances for application in next‐generation semiconductors. Materials with high cohesive energy can be used as indicators of EM performance as they can slow down the EM as a barrier to void formation and self‐diffusion.

### 2.3 Multilayered Interconnect Structures Process

**Figure 6:**
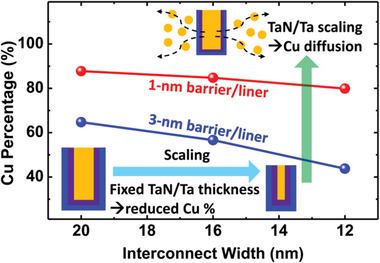
Changes in the Cu occupancy percentage of the entire interconnect cross‐sectional area with scaling. Note that this is not a volume fraction. The reduced Cu footprint further aggravates the interconnect problems. Reducing the thickness of the conventional barrier/liner raises problems in Cu diffusion. Reproduced with permission.^[^ ^43^ ^]^ Copyright 2020, American Institute of Physics publishing.

This section will discuss the issues that arise from fundamental multilayer interconnection structures as the CD decreases. Because a particular volume of barrier and liner layer is required for device reliability and process procedure, the conductor has insufficient space to drive the current (**Figure** **6**) effectively. Therefore, more compact layers are required to achieve superior current conduction while the downscaling continues. In recent nodes, the minimum thickness of the liner/barrier layer required for the Cu damascene process has reached a point where it can no longer be reduced. It leads to a sharp decrease in the volume fraction occupied by the conductors in the via or trench structure, accelerating resistivity size effects and EM and creating process issues that make super‐filling difficult. In particular, the vias between metal lines have CDs as small as 12–14 nm, letting via resistance be a significant hurdle to overcome for attaining superior interconnect performance levels in next‐generation technology nodes. Furthermore, as scaling continues, the barrier layer, possessing a relatively higher resistivity than the conductor and the multilayer structure formed perpendicular to the current path, increases the contact resistance and contributes to the entire device resistance.^[^ ^128^ ^]^ In addition, the narrowed area leads to a pinch‐off by forming a defect while filling the gap due to the non‐uniform application of the Cu seed layer via conventional PVD.^[^ ^129^ ^]^

In 1997, IBM announced Cu as a new interconnect material for 220‐nm node technology, replacing the conventional Al conductor material.^[^ ^130^ ^]^ Cu has several beneficial properties, such as lower electrical resistivity, higher EM, higher stress‐migration resistance, and higher melting point than Al, making it a more suitable modern internal wiring material.^[^ ^122^, ^131^, ^132^, ^133^ ^]^ However, another problem has arisen, as discrete patterning and deposition methods should be applied to Al and Cu. For instance, Cu materials cause additional problems, such as the diffusion of Cu into the Si and Si‐based insulating layers at relatively low temperatures, corrosion of Cu during the chip fabrication process due to the absence of a self‐passivized oxide layer, and poor adhesion between Cu and the insulating layers, resulting in the degradation of Cu‐based devices.

**Figure 7:**
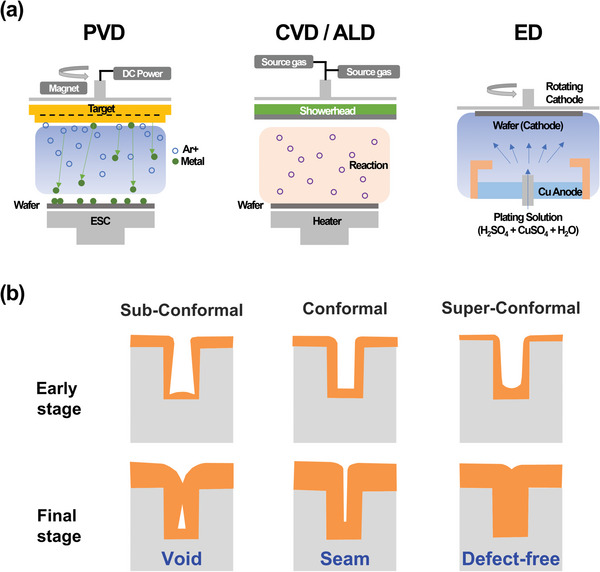
a) Schematic of various deposition methods. b) Profiles of sub‐conformal, conformal, and super‐conformal Cu gap‐filling.

Therefore, to ensure that Cu interconnects successfully function as internal wires, a series of elaborate processes should be done thoroughly, including advanced methods like the damascene process and various other deposition methods (**Figure** **7**a).^[^ ^134^ ^]^ First, a low‐*k* dielectric layer is deposited on the Si wafer to reduce the capacitance and delay of the chip. The via and trench are subsequently formed by etching the insulating material using a patterning process. Then, a thin film of barrier materials such as Ta/TaN,^[^ ^135^, ^136^, ^137^, ^138^ ^]^ W_2_N,^[^ ^139^, ^140^ ^]^ TiN,^[^ ^141^, ^142^, ^143^ ^]^ TiC,^[^ ^144^, ^145^ ^]^ TiW,^[^ ^146^ ^]^ TaSiN,^[^ ^147^, ^148^, ^149^, ^150^, ^151^ ^]^ usually the composites of transition metals and N, C, or Si, are inert to both conductors and insulators at relatively high temperatures and deposited on top employing PVD or CVD.^[^ ^152^, ^153^, ^154^, ^155^ ^]^ However, suppose the conductor metal atoms diffuse and penetrate the dielectric layer. In that case, they agglomerate inside the dielectric layer and act as a deep trap site or shortcut, causing dielectric leakage and eventually lowering the reliability of the entire device. Additionally, the Cu seed layer is deposited before the next step, as Cu has a low conductivity and poor nucleation behavior on the barrier layer (usually Ta/TaN, which is the most frequently used liner/barrier layer of the past three decades).^[^ ^156^, ^157^ ^]^ Subsequently, the ED process of Cu can fill the damascene features, terminating the process by CMP of the over‐plated Cu.

The most crucial thing is defect‐freely filling the Cu in the trenches during damascene processes within this cycle. Metallization based on ED has been a practical method for a long time for Cu interconnect metallization of IC chips. It is a time‐ and cost‐efficient process that can fill the high‐aspect‐ratio trenches without voids and seams. As widely known, PVD is unsuitable for filling the vias with a high aspect ratio owing to its strong shadow effect.^[^ ^158^ ^]^ Although appropriate precursors allow high‐purity conformal filling by CVD, an alternative technique is desirable because chemical precursors are often toxic and require high temperatures.^[^ ^159^, ^160^ ^]^ Figure 7b shows a representative scheme for filling metal into the trench structure. A sub‐conformal profile refers to the formation of voids inside the trench due to intensive metal deposition at the trench entrance.^[^ ^161^ ^]^ It dramatically reduces the reliability of the wiring. This morphology generally appears in PVD, and in the case of ED, it appears when the mass transfer of the metal ion is dominant. A conformal profile shows when the deposition rate is the same on every side, and a seam is formed when the metal growing from the sides of the trench meets.

A super‐conformal profile, also called super‐filling, has no defects inside the trench and is characterized by the formation of bumps at the trench entrance. This morphology can be achieved by selectively increasing the ED rate at the bottom of the trench by appropriately controlling the adsorption of additives and surface coverage. They can be classified into accelerators and suppressors based on their effect on the ED rate. In general, Cu plating accelerators are organic materials containing disulfide (—S—S—) or thiol (—SH) bonds, which supply electrons to Cu^2+^ in solution and form a complex to increase the plating speed.^[^ ^162^, ^163^, ^164^, ^165^ ^]^ However, the suppressors slow the deposition rate by adsorbing onto the seed layer, preventing access to Cu ions. In addition, the chloride ions (Cl^−^) are pre‐adsorbed on the Cu surface to facilitate further adsorption of polyethylene glycol, thereby interfering with the charge transfer reaction between the Cu ions and the electrode.^[^ ^166^, ^167^ ^]^ Typically, Cu damascene super‐filling incorporates an additive combination of polyethylene glycol and bis(3‐sulfopropyl) disulfide. As the technology node progresses, new alkali‐based plating solutions are being developed, and alternative materials are emerging to form Cu within a narrow area efficiently.^[^ ^129^ ^]^

### 2.4 Projection

Herein we have discussed three problems encountered when reducing the area of the BEOL to the nanoscale. First, resistance size effect problems arise as scaling accelerates in conductor metals. Nanoscale electron scattering can be improved by substituting a material that is less susceptible to size effects, considering the correlation between the material surrounding the conductor and grain engineering. Second, intrinsically strong atomic bonds in a conductor can significantly resist atomic migration regarding reliability. Therefore, we can solve the interconnect industry problems by introducing a new material with a small resistivity size effect and inherently strong bonds. Third, the multilayer structure required to route the conductor material aggravates the first two problems and creates process problems.

Moreover, the transition to new materials requires changes in the processes and designs used in the industry. The following sections discuss new materials to address these three problems comprehensively. The first alternative is the introduction of a new barrier/liner to sustain the current Cu wiring. Subsequently, in preparation for the continued development of the technology node, we discuss switchable central conductor materials.

## 3 Next‐Generation Interconnect Materials

### 3.1 Advanced Barrier and Liner Layers

Several tens of kilometers of Cu wiring have recently been embedded in the 5‐nm node system on chip (SoC) technology. Although research is being actively conducted on materials to replace Cu to solve the interconnect scaling problem, the upper part of the chip still requires a large amount of Cu. Furthermore, the industry will not allow the affordable and standardized damascene process to disappear in future generations. Therefore, research on barriers and liners required for Cu wiring is still ongoing.^[^ ^43^, ^168^, ^169^, ^170^ ^]^ In this section, we discuss studies on replacing double barrier/liner layers with monolayered materials.

New ideal barrier/liner materials should have the following characteristics to solve the previously mentioned problems: 1) outstanding adhesion capacity with the Cu metal and dielectric layer, 2) outstanding conductivity for direct Cu ED, 3) immiscibility with Cu, 4) ability to prevent Cu diffusion at high temperatures, and 5) capability of facile and uniform deposition of an ultra‐thin film on the dielectric layer. Therefore, under these requirements, suitable candidates as future barrier materials would be Pt group metal (PGM)‐based materials, 2D materials, self‐assembled molecular layers (SAMs), and high‐entropy alloys (HEAs).

#### 3.1.1 Metallic Barriers for Metallization

Ru is currently a promising conductor material and the most attractive barrier material.^[^ ^171^, ^172^ ^]^ Ru has a high melting temperature with outstanding adhesion to Cu metal owing to its excellent wettability but insignificant solubility with Cu. From a thermodynamic perspective, Ru has an excellent affinity with Cu, enabling underpotential plating, which can obtain a Cu layer with high continuity in the initial deposition phase.^[^ ^173^ ^]^ In addition, its electrical resistivity is 7.1 µΩ cm,^[^ ^174^ ^]^ much lower than that of Ta (13 µΩ cm), permitting the successful direct electroplating of Cu. However, similar to other barrier materials, the diffusion‐limiting efficiency of pure Ru is highly dependent on the layer thickness and heat exposure time. For example, a 22‐nm‐thick layer can withstand temperatures above 450 °C for 10 min, whereas a 5‐nm‐thick layer cannot withstand a temperature of 300 °C for the same duration.^[^ ^174^, ^175^ ^]^ Its columnar grain structure provides a path for Cu diffusion, allowing Cu atoms to penetrate the Si substrate in the Cu/Ru/Si system.

**Figure 8:**
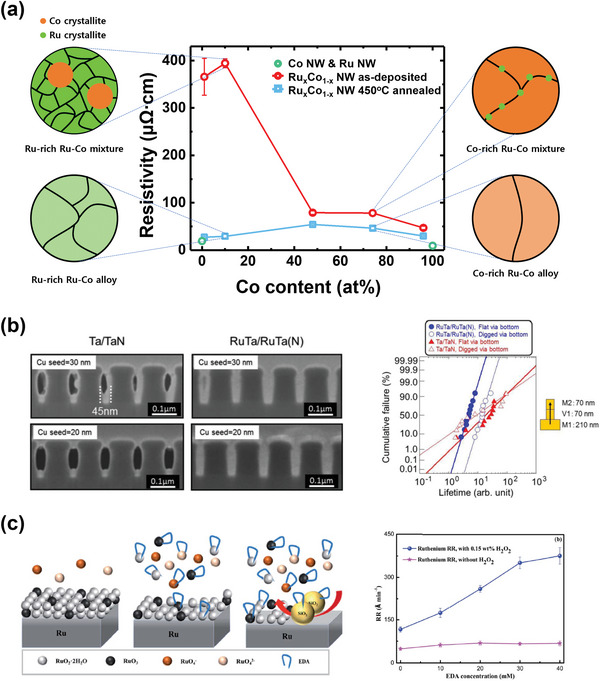
Studies on Ru‐based barrier metals to replace the conventional TaN/Ta structure. a) The electrodeposited Ru_1−_ *_x_* Co*_x_* (0 < *x* < 1) nanowires (diameter = 130 nm) have lower resistivity than conventional TaN (130‐nm‐thick thin film *ρ*_TaN_ = 250–400 µΩ cm)^[^ ^181^, ^182^ ^]^ after annealing at 450 °C for 1 h. Reproduced with permission.^[^ ^54^ ^]^ Copyright 2022, Elsevier. b) Cross‐sectional scanning electron microscopy (SEM) images of the 45‐nm‐wide trenches with Ta/TaN and RuTa/RuTa(N) barrier layers (left). Statistical distribution of via EM lifetime at the M1 trench (width: 210 nm), V1 via (diameter: 70 nm), and M2 trench (width: 70 nm) (right). Reproduced with permission.^[^ ^183^ ^]^ Copyright 2016, The Electrochemical Society. c) Schematic of the CMP mechanism of H_2_O_2_‐based slurries on Ru (left). The removal rate of Ru as a function of EDA concentration (right). Reproduced with permission.^[^ ^199^ ^]^ Copyright 2022, Royal Society of Chemistry.

The doping or alloying of foreign elements helps solve various problems at the GBs. This strategy can improve the barrier properties by forming a GB stuffing effect or an amorphous structure, expecting a low resistivity. Sputtering the Ru thin film layer in an N_2_ atmosphere helps develop an amorphous structure, destroying the columnar‐shaped grains and postponing the formation of Ru silicide,^[^ ^176^ ^]^ which initiates failure and further promotes Cu diffusion by creating Cu silicide.^[^ ^177^ ^]^ According to the X‐ray diffraction patterns, the failure temperature in the Cu/Ru‐N/Si system is 900 °C, whereas that in the Cu/Ru/Si system is 700 °C.^[^ ^176^ ^]^ The amorphous 5‐nm Ru*_x_* Cr_1−_ *_x_* film did not show significant interdiffusion in the Cu/Ru*_x_* Cr_1−_ *_x_*/Si structure for 30 min at 650 °C because of its high thermal stability.^[^ ^178^ ^]^ The 5‐nm Ru*_x_* Mo_1−_ *_x_* film exhibited a 100‐fold improvement in current leakage compared to the pure Ru film. The thermal stability increased by 175 °C, resulting in a better barrier effect at the annealing temperature of 725 °C.^[^ ^179^ ^]^ The 25‐nm Ru*_x_* W_1−_ *_x_* thin film showed no Cu penetration into Si at 650 °C and exhibited a lower wetting angle (43°) than that of Cu (123°) on Ta substrates.^[^ ^180^ ^]^ In the Cu/Ru*_x_* Co_1−_ *_x_* (*x* < 0.4)/NiSi*_y_*/Si structures of various compositions synthesized by the electroplating method, Cu diffusion was not observed for 10 min at 400 °C but was observed for the Cu/Ru/NiSi*_y_*/Si structure under the same conditions.^[^ ^52^ ^]^ Electroplated Ru*_x_* Co_1−_ *_x_* (0 < *x* <100) nanowires show lower resistivity than those of TaN with the same diameters in all compositions (**Figure** **8**a).^[^ ^54^, ^181^, ^182^ ^]^ In the RuTa/RuTa(N) barrier structure, RuTa was strongly oriented toward Ru(002), and the lattice mismatch between Ru(002) and Cu(111) was lower than that between Ta(110) and Cu(111), demonstrating excellent wettability. This structure exhibits low resistance and long EM life expectancy owing to its outstanding wettability and filling properties (Figure 8b).^[^ ^183^ ^]^

The Ru barrier/liner is uniformly applied in an actual trench structure by CVD, ALD, or even the ED method.^[^ ^184^, ^185^, ^186^, ^187^, ^188^ ^]^ Ru was deposited by electroless plating from an alkali solution inside a through silicon via (TSV) with an aspect ratio of 11 covered with Ta.^[^ ^188^ ^]^ Conformal plating was then achieved in the TSV regions using an alkaline Cu bath. The biggest obstacle in applying Ru‐based barriers/liners is the CMP process. The high strength, hardness, and low chemical reactivity of Ru reduce the efficiency of traditional CMP slurries. Inadequate slurry control can also produce harmful oxide byproducts. At low pH and high electrochemical potential, Ru is prone to form toxic and volatile RuO_4_.^[^ ^189^ ^]^ Therefore, oxidizing agents, such as potassium perchlorate (KClO_4_), potassium permanganate (KMnO_4_), sodium hypochlorite (NaClO), potassium bromate (KBrO_3_), potassium persulfate (K_2_S_2_O_8_), potassium periodate (KIO_4_), and hydrogen peroxide (H_2_O_2_), have been screened.^[^ ^190^, ^191^, ^192^, ^193^, ^194^, ^195^, ^196^ ^]^ The presence of IO_4_^−^ ions in the slurry promotes the formation of mechanical structures and porous oxide layers on the Ru surface. It accelerates surface corrosion, thereby increasing the Ru removal rate.^[^ ^197^ ^]^ However, the significant potential gap between Ru and Cu can cause severe galvanic corrosion of Cu. In contrast, H_2_O_2_ creates a negligible corrosion potential difference between Ru and Cu, forming a smooth and dense Ru surface.^[^ ^198^ ^]^ In addition to oxidizing agents, complexing agents like ethylenediamine (EDA) are being investigated because they can accelerate material removal by chelating metal ions to create soluble species in CMP (Figure 8c).^[^ ^199^ ^]^

#### 3.1.2 2D Material Barriers

**Figure 9:**
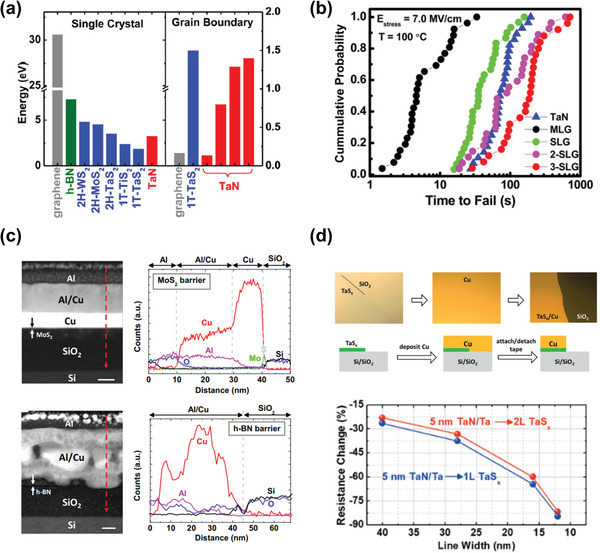
a) The barrier energies for Cu diffusion of various 2D materials with single crystals and grain boundaries, obtained by first‐principles DFT calculations. Reproduced with permission.^[^ ^43^ ^]^ Copyright 2020, American Institute of Physics publishing. b) Cumulative distribution of the TDDB lifetime with single‐layer graphene (SLG), 2‐SLG, 3‐SLG, multilayer graphene (MLG), and 4‐nm TaN stressed at 100 °C and 7.0 MV/cm electric field. Reproduced with permission.^[^ ^204^ ^]^ Copyright 2015, American Chemical Society. c) Cross‐sectional scanning TEM images and line energy‐dispersive X‐ray spectroscopy profiles of directly grown MoS_2_ (top) and transferred h‐BN (bottom). Reproduced with permission.^[^ ^208^ ^]^ Copyright 2017, Nature publishing group. d) Adhesion tests using the tape method (top). After depositing TaS*_x_* on half of the Si/SiO_2_ substrate, an 80‐nm Cu layer was deposited. When the tape is attached and detached, Cu remains where TaS*_x_* is present, indicating good adhesion to Cu. Resistance changes as a function of line width when 5‐nm TaN/Ta is replaced with one or two layers of TaS*_x_* (bottom). As the line width decreases, the thinning of the barrier/liner thickness results in a tremendous resistance change. Reproduced with permission.^[^ ^210^ ^]^ Copyright 2019, Wiley‐VCH.

Compared to conventional materials with three‐dimensional nature, 2D materials have great potential for achieving a sub‐nm barrier/liner in advanced technology nodes.^[^ ^200^ ^]^ Similar to alternative metallic materials, first‐principles density functional theory (DFT) calculations provide initial insights into the electronic structure of the interface between Cu and various 2D materials to block Cu diffusion (**Figure** **9**a). Comparing the calculated energy barrier of 2D materials with the experimentally extracted values for TaN allows for rapidly identifying promising candidates. While graphene, hexagonal boron nitride (h‐BN), and some sulfides are expected to demonstrate superior barrier properties compared to TaN in crystals that are free of defects,^[^ ^43^ ^]^ the presence of a high defect and impurity densities during growth can create pathways for Cu diffusion, ultimately leading to a decrease in energy barrier. Nevertheless, since their inherent atomically thin body thicknesses are attractive, we introduce several approaches and challenges for utilizing 2D materials in BEOL processes.

First, graphene is a one‐atom‐thick carbon film with a highly compact 2D structure impenetrable by any metal atoms. In addition, graphene is thermally and chemically stable and has high thermal and electrical conductivities. Therefore, some researchers have examined the ability of CVD‐grown graphene as an adequate barrier material to prevent Cu diffusion.^[^ ^201^, ^202^, ^203^ ^]^ Meanwhile, as discussed above, because grain size control is crucial in managing Cu diffusion, some studies have examined this by alternating the number of graphene layers.^[^ ^202^ ^]^ The 0.35‐nm‐thick graphene monolayer demonstrates a similar average time to failure compared to 4‐nm TaN, which improves as the number of graphene layers increases (Figure 9b).^[^ ^204^ ^]^ While increasing the grain size, the oxidation rate decreased more rapidly as the graphene sheets increased from one to four, with 240 min of annealing at 200 °C. In addition, there are more 2D materials suitable for Cu diffusion barriers, such as h‐BN and transition metal dichalcogenides (TMDs). Although h‐BN has a problem with arresting the direct ED of Cu on top due to its resistive nature and Cu intercalation into h‐BN, many studies have demonstrated that h‐BN thin films could serve as a long‐term oxidation‐proof barrier layer for Cu by hindering the diffusion of Cu into the dielectric layer (Figure 9c).^[^ ^205^, ^206^, ^207^, ^208^ ^]^ MoS_2_, WS_2_, MoSe_2_, and WSe_2_, examples of 2D TMDs, are also suitable candidates as barrier materials because they effectively suppress Cu penetration.^[^ ^209^ ^]^ The 1.5‐nm TaS_2_ exhibits better diffusion barrier properties than 3‐nm Ta and is similar to 2‐nm TaN. It makes it possible to replace the 5‐nm TaN/Ta bilayer with 3‐nm TaS_2_, thus significantly increasing the Cu occupancy ratio in the trench structure and reducing the line resistance of the interconnects (Figure 9d).^[^ ^210^ ^]^

Several challenges remain in the application of 2D materials to state‐of‐the‐art BEOL processes. A primary task is to overcome the differences between the conventional PVD‐based TaN/Ta deposition method and the current CVD‐based 2D material synthesis, which requires adequate modifications to the design and process. PVD has a relatively low process temperature and less substrate dependency, whereas CVD can only grow 2D materials at high temperatures and on specific substrates. For example, graphene grows preferentially on metals rather than dielectrics, whereas TMDs grow on dielectrics.^[^ ^211^, ^212^ ^]^ The thermal energy to dissociate the CVD precursor is generally above 700 °C, significantly higher than the BEOL process temperature below 400 °C. A high process temperature damages the dielectric around the wiring, ultimately affecting reliability. Plasma‐enhanced CVD (PECVD) or metal‐organic CVD (MOCVD) for 2D materials synthesis have been studied to lower growth temperatures.^[^ ^203^, ^213^, ^214^, ^215^ ^]^ However, 2D material deposition at low temperatures has poor barrier performance because the nanocrystalline or amorphous form produces a small grain size.^[^ ^43^, ^214^, ^216^ ^]^ The plasma growth process can degrade the low‐dielectric properties.

Although current CVD methods limit the grain size, it may become a future research focus because of the possibility of achieving single‐layer 2D materials with inherently large grain sizes owing to their material properties. In addition to process issues, the conductivity anisotropy of 2D materials must be considered for application to actual structures. The conductivity of most 2D materials has only been discussed concerning in‐plane resistivity and rarely concerning out‐of‐plane resistivity. Compared to the typical room‐temperature resistivity of 1T‐TaS_2_ (space group P‐3m1) and 2H‐TaS_2_ (space group P‐6m2), which are ≈300 and 150 µΩ cm, respectively, their out‐of‐plane resistivities reach 7 × 10^5^ and ≈5000 µΩ cm, respectively.^[^ ^217^, ^218^ ^]^ This can be a significant bottleneck when the current flows from the upper‐level M*_x_*_+1_ through “via” to the lower‐level M*_x_*. Therefore, for the 2D material to be used as an interconnect material, it is necessary to develop a technology that can deposit parallel to the electron movement direction in a vertical via or trench structure, or develop a material that can satisfy the trade‐off according to thickness. In other words, a material with the thinnest thickness possible minimizes vertical resistance while having barrier properties. Significantly more studies are required until the increased Cu volume resulting from the reduced thickness of the 2D materials‐based barrier/liner layer, which is thinner than the conventional structure at the ultra‐scale, becomes advantageous.

#### 3.1.3 Self‐Assembled Molecular Layer

The spontaneous organization of molecules under thermodynamic or kinetically controlled conditions forms self‐assembled molecular layers (SAMs) with long‐range order. Organic SAMs of sub‐nanometer‐sized molecules comprise a head group, an alkyl chain, and a terminal functional group. They can be customized to achieve the desired surface chemistry, structure, and thickness. The head groups are bound to the monolayer domain, and the end groups determine the properties of the SAM‐modified surface. The nature of the terminal group of SAMs can significantly influence the barrier effect against Cu diffusion. SAMs built with P‐containing molecules (P‐SAMs) can form Cu‐P complexes during annealing. A 1.7‐nm‐thick P‐SAM diffusion barrier has a lifetime equivalent to a 20‐nm‐thick Ta film.^[^ ^219^ ^]^ In addition, among the P, C, and N‐SAMs, the time‐to‐breakdown of the dielectric layer of P‐SAM‐modified SiO_2_ was ten times longer than that of bare SiO_2_, whereas C‐ and N‐SAMs showed inconspicuous improvements.^[^ ^220^ ^]^ Molecules with aromatic rings, such as zinc porphyrin, can provide a steric hindrance to Cu diffusion.

**Figure 10:**
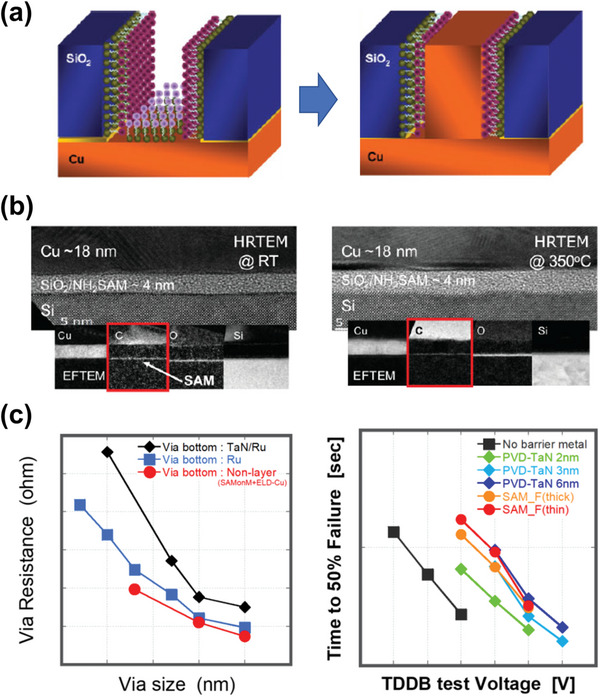
a) Schematic diagram of the Cu bottom‐up fill mechanism using SAM. The selected ability of the head and terminal groups of SAM can give the desired functionality. b) NH_2_‐SAM exhibits excellent Cu diffusion barrier properties at room temperature and an annealing temperature of 350 °C. Reproduced with permission.^[^ ^225^ ^]^ Copyright 2010, Wiley‐VCH. c) Results of via resistance and TDDB tests conducted on Cu interconnects as a replacement for conventional barrier/liner materials. The decrease in via resistance is owing to the increase in Cu volume, and the TDDB test results indicate that SAMs exhibit better resistance than PVD‐TaN 3 nm regardless of the thickness of the SAM layer. Reproduced with permission.^[^ ^226^ ^]^ Copyright 2022, Institute of Electrical and Electronics Engineers.

In addition, the electronegativity of the zinc atom at the center of the porphyrin core further inhibits Cu diffusion.^[^ ^221^ ^]^ In other studies, —NH_2_ terminated SAM was considered the most promising SAM‐based barrier material for Cu diffusion.^[^ ^222^, ^223^, ^224^ ^]^ Cu/NH_2_‐SAM/SiO_2_ remained stably bonded up to 600 °C and functioned well as a barrier layer. The high‐temperature resistance of NH_2_‐SAM, based on 3‐aminopropyltrimethoxysilane (APTMS), suggests a novel mechanism for the metallization method (**Figure** **10**).^[^ ^225^ ^]^ In the via structure, where the lower layer Cu is deposited, a sacrificial SAM (S‐SAM) with a head group (i.e., —SH, thiol) selectively binds to Cu, and an inactive terminal group (i.e., —CH_3_, methyl) bonds Cu at the bottom of the vial. Next, a barrier SAM (B‐SAM) with a head group (i.e., —Si(OCH_3_)_3_, trimethoxysilane) binds to the surrounding low‐k dielectric, and a terminal group (i.e., —NH_2_, amine) binds only to the Cu that is attached to the outer wall of the vial. The S‐SAM is detached within the process temperature, and the B‐SAM, which replaces the existing barrier/liner layer, is attached to the dielectric. Then, the upper layer of Cu and vias can be filled through a bottom‐up‐fill method using the lower layer of Cu as an electrode. Replacing the Cu barrier metal with a SAM material possessing Cu barrier properties and adhesion to Cu and dielectric can reduce the via resistance in Cu interconnections. This approach increases the volume of Cu while minimizing the number of process steps required.^[^ ^226^ ^]^ The metallization mechanism used for SAMs in the wiring process has been discussed until recently. It is a typical method that can be used to achieve reductions in both via resistance and fallibility.^[^ ^227^ ^]^

#### 3.1.4 High‐Entropy Alloys

High‐entropy alloys (HEAs) are customarily fabricated by alloying more than five metallic elements in equal or relatively large proportions. HEAs have several unique characteristics, such as high entropy,^[^ ^228^, ^229^ ^]^ lattice distortion,^[^ ^230^, ^231^, ^232^ ^]^ sluggish diffusion,^[^ ^233^, ^234^, ^235^ ^]^ and cocktail effects,^[^ ^236^ ^]^ which result in excellent properties such as high thermal, chemical, and mechanical stabilities, interdiffusion resistivity, and hardness. Because of these characteristics, HEAs can easily form amorphous structures or solid solutions to suppress Cu interdiffusion while different atoms of various sizes are mixed.^[^ ^237^, ^238^, ^239^ ^]^ For example, the DC magnetron sputtering of a multi‐component AlCrTaTiZrRu/(AlCrTaTiZrRu)N_0.7_ double‐layer remained amorphous with no Cu‐silicide compounds found on the Si substrate up to the annealing temperature of 800 °C. However, the sheet resistance dramatically soared from 0.070 to 243 Ω sq^−1^ when the annealing temperature was increased from 800 to 900 °C, indicating the total failure of the double‐layer. Since the concept of HEA was first reported in 2004,^[^ ^240^ ^]^ it has attracted attention for the effective prevention of Cu diffusion; however, there has been limited discussion on its resistance and actual process application. An active investigation of HEAs with better properties based on advanced material designs is expected.

### 3.2 Alternative Conductor Materials

If the technology node continues to evolve, the lower layers of the interconnect will reach a limit where Cu can no longer be used. In the most obvious way, the industry is trying to advance the technology node by introducing new conductor materials. In the previous sections, we discussed the electrical resistivity of metalized conductor lines and the reliability owing to the movement of metal atoms. The effects of resistivity magnitude and the atom movement phenomenon are related to the intrinsic properties of interconnect metals. Early screening approaches to displace Cu relied on heuristic proxies, such as bulk resistance and melting point. Recently, a screening method based on the ab initio calculations of the Fermi surface has emerged.^[^ ^39^, ^45^ ^]^ In semiclassical equations (Equation (4)), the contribution to resistivity by surface and GB scattering is proportional to *ρ*_0_  ×  *λ*, which is related to the electronic structure of the metal. If different metals have identical diameters, surface states, and grain structures, the resistivity contribution will be determined by the electronic structure of the metal (Equation (4)). In other words, the *ρ*_0_  ×  *λ* product, which leads to low bulk resistance and sensitivity to finite size effects, can be used as a “figure of merit (FOM)” parameter for resistivity scaling because it guarantees low resistance of scaled interconnect structures. The cohesive energy can be used as a proxy for both EM and the requirements for diffusion barriers.^[^ ^241^ ^]^

**Figure 11:**
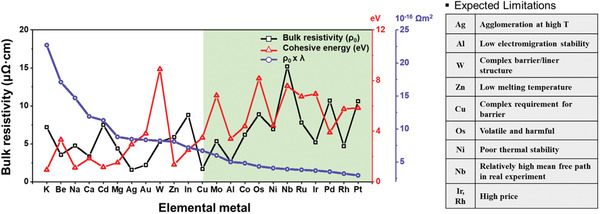
Candidate metals screened by first‐principle calculations and expected limitations for IC implementation. All elemental metals, except Al, in green shades, are expected to show a low increase rate in resistivity and have higher cohesive energy than Cu. Co, Mo, and Ru are appropriate for next‐generation interconnect materials considering process issues. The calculated values used.^[^ ^39^, ^45^, ^242^ ^]^ Reproduced with permission.^[^ ^39^ ^]^ Copyright 2016, American Institute of Physics publishing. Reproduced with permission.^[^ ^45^ ^]^ Copyright 2017, American Institute of Physics publishing. Reproduced with permission.^[^ ^210^ ^]^ Copyright 2005, John Wiley & Sons.

**Figure** **11** shows the cohesive energy and the *ρ*_0_  ×  *λ* product, based on the bulk electronic structure predicted by first‐principles DFT calculations for 23 conductive elemental metals.^[^ ^39^, ^45^, ^242^ ^]^ For body‐centered‐hexagonal (*hcp*) and body‐centered‐tetragonal (*bct*) structures, which have different FOM values depending on the direction of the crystal structure, transport parallel to the hexagonal/tetragonal axis with lower values are indicated. Notably, Pt, Rh, and Ir have FOM values that are 2.4, 2.1, and 1.8 times smaller than those of Cu. Regarding reliability, Co, Ni, Mo, Nb, and PGMs with high cohesive energies are promising and are expected to show better potential than Cu. However, only Co and Ru are highlighted as promising materials when considering additional parameters, such as process and material cost. Although they show bulk resistivity values 3–5 times greater than that of Cu, resistivity reversal is predicted to occur near 10 nm because of their low FOM values. In particular, Ru has been the most researched as the best substitute for Cu and Co.

**Figure 12:**
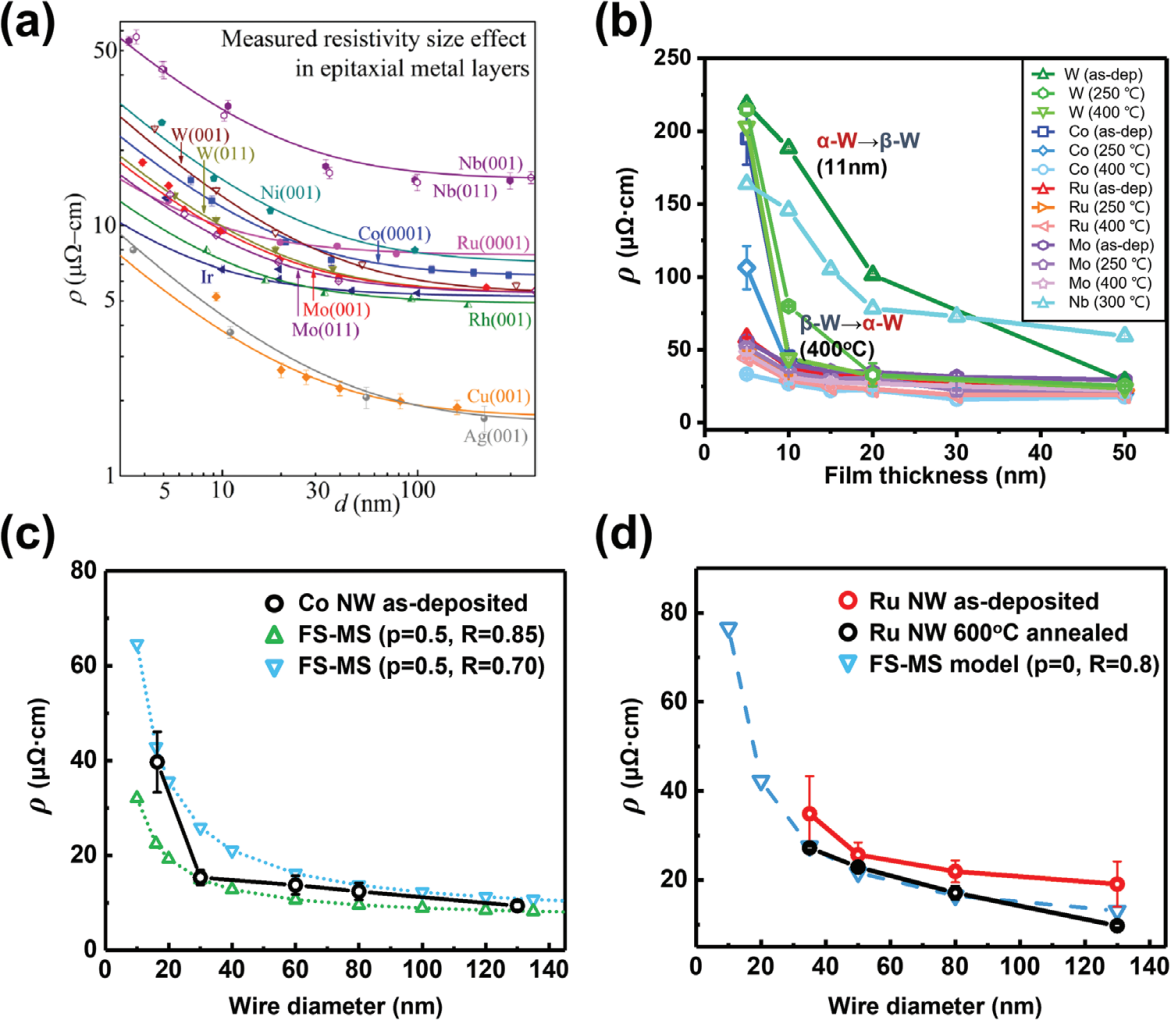
Variation in resistance due to scaling of various metal elements measured by the 4‐point probe method. A collection of 2D film resistivity values of a) epitaxial metal layers and b) polycrystalline metal layers. Nb was expected to have low resistivity scaling by FOM but exhibits relatively high resistivity. Cu shows the lowest resistivity value in the absence of GB scattering but has a higher line resistance in the actual structure when a barrier/liner layer is added. a) Reproduced with permission.^[^ ^49^ ^]^ Copyright 2020, American Institute of Physics publishing. Changes in resistivity as a function of the diameter of electrodeposited c) Co and d) Ru nanowires. The crystal structure and dimension of the metal affect the nanoscale electron scattering, dramatically increasing resistivity. c) Reproduced with permission.^[^ ^53^ ^]^ Copyright 2020, Elsevier. (d) Reproduced with permission.^[^ ^54^ ^]^ Copyright 2022, Elsevier.

Practical comparisons between the experimental quantification and theoretically calculated values, approximated by semiclassical BTE formalism in resistivity scaling for different metals, show distinct differences due to several variables. The resistivities of nanoscale conductors depend on surface roughness,^[^ ^82^, ^243^, ^244^ ^]^ crystal orientation,^[^ ^53^, ^245^ ^]^ type of process,^[^ ^48^ ^]^ chemistry,^[^ ^243^ ^]^ GB density,^[^ ^246^ ^]^ structure,^[^ ^54^, ^93^, ^99^ ^]^ and orientation distribution.^[^ ^91^, ^247^ ^]^ For example, the predicted values from the Cu simulation and experimentally obtained effective EMFP were the same as 39 nm. However, the experimental value of Nb is approximately 10 times larger than the simulation value, making it unsuitable for next‐generation wiring materials in terms of the size effect (**Figure** **12**a,b).^[^ ^47^, ^248^ ^]^ Moreover, because Equation (4) includes three unknown parameters, viz. *λ*, *p*, and *R*, which behave independently, it is difficult to derive an accurate resistivity value, even if it is determined experimentally. Thus, idealized material systems, such as epitaxial metal layers (*R* = 0), have been employed in several studies to quantify the resistive scaling for specific metals and determine the mean free path or surface scattering parameter.^[^ ^244^, ^245^, ^249^, ^250^, ^251^ ^]^ However, these studies are restricted to 2D thin films and provide ideal results with several approximations and assumptions. The semiclassical model based on Boltzmann transport fits well when the thickness of the thin film is larger than the EMFP but is underestimated when *d* < 20 nm. In addition, a new quantum mechanical model is being developed because the existing classical model completely fails below 10 nm.^[^ ^64^, ^66^ ^]^ The actual wiring structure is similar to a 1D line shape. There is a definite difference between the 2D thin‐film when considering the crystal morphology and surface scattering frequency due to the geometric structure (Figure 12c,d).^[^ ^53^, ^54^, ^252^, ^253^ ^]^ Nevertheless, the semiclassical model approximation and cohesive energy provide a clue for promising next‐generation wiring materials and allow further research. Significant efforts are being devoted to verifying the resistance scaling by fabricating Co, Ru, intermetallic compounds, and MAX phases, highlighted as promising materials to form actual wiring structures. These are polycrystalline line types, including liner and barrier structures, and include the EM and TDDB performance results.

#### 3.2.1 Cobalt

**Figure 13:**
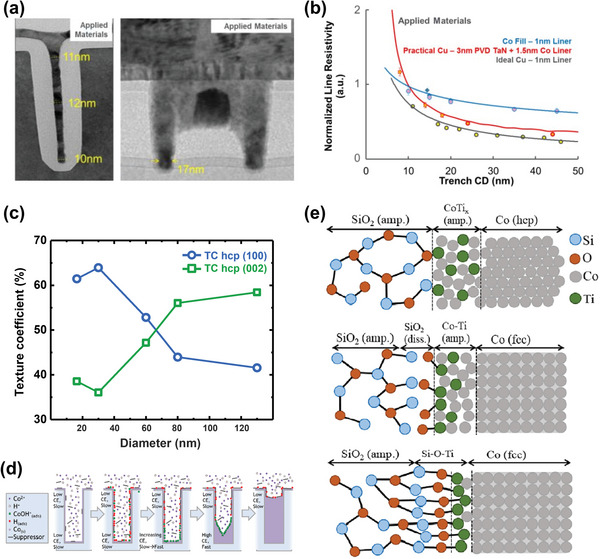
a) Cross‐section TEM images of Co void‐free gap‐fill of 10‐nm CD trenches and 17‐nm via bottom CD dual‐damascene structures by Applied Materials. b) Crossover of the line resistivity of Co and Cu interconnects according to trench CD. Co with a 1‐nm liner (blue), Cu with a 3‐nm barrier and 1.5‐nm liner (red, practical Cu case), and Cu with a 1‐nm liner (black). Reproduced with permission.^[^ ^255^ ^]^ Copyright 2017, Institute of Electrical and Electronics Engineers. c) The texture coefficient (TC) dependence of electrodeposited Co nanowires on the diameter. A change in the crystal orientation contributes to a change in resistivity. Reproduced with permission.^[^ ^53^ ^]^ Copyright 2020, Elsevier. d) Co super‐conformal filling mechanism due to a pH gradient inside the trench. CE = current efficiency. Reproduced with permission.^[^ ^263^ ^]^ Copyright 2018, The Electrochemical Society. e) Schematic illustration of the reaction behavior of Co/CoTi*_x_*/SiO_2_ according to the annealing temperature: as‐deposited (top), annealed at 500 °C (middle), and at 700 °C (bottom). The dotted line indicates the interface between the layers; amp. = amorphous structure; diss = dissociated state. Reproduced with permission.^[^ ^269^ ^]^ Copyright 2018, Elsevier.

Engineers have investigated Co as an alternative to middle‐of‐line materials because of its high cohesive energy and the low EMFP with less sensitivity to the size effects. The theoretically predicted FOM for Co along the *z*‐axis of the hexagonal‐close‐packed (*hcp*) structure is 4.82 × 10^−16^ Ω m^2^, while that of body‐centered cubic (*bcc*) W and face‐centered‐cubic (*fcc*) Cu are 8.20 × 10^−16^ and 6.70 × 10^−16^ Ω m^2^, respectively_._ In 2018, Intel introduced Co as a wire material in the lowest two layers among 12 metal interconnects based on the ED process in the 10‐nm node technology process.^[^ ^254^ ^]^ Although the resistance of the Co line at the 20‐nm line CD of the same liner/barrier structure is larger than that of Cu,^[^ ^186^ ^]^ the trade‐off between high EM resistance and lower vertical contact resistance successfully led to material conversion to Co.^[^ ^254^ ^]^ Moreover, as scaling continues and the trench CD becomes below 14 nm, a resistance crossover occurs between practical Cu and Co interconnects with thinner TiN barriers (thickness = 1 nm) owing to the high EM resistance (**Figure** **13**a,b).^[^ ^255^ ^]^

In addition to the resistive scaling aspect, Co is an attractive replacement player because of its low process cost and bottom‐up filling, which is highly compatible with industry‐standard deposition tools. Depending on the process conditions, ED can easily control the factors that play an essential role in wire scalings, such as crystal structure, orientation, and grain size.^[^ ^53^, ^54^, ^256^, ^257^ ^]^ Co and Ru, which have an *hcp* structure, have different MFPs parallel and perpendicular to the basal plane.^[^ ^39^ ^]^ Co nanowires grown by a bottom‐up fill method in an aqueous ED bath can change their crystal structures from *fcc* to *hcp* depending on various parameters, such as the applied voltage, pH, and temperature.^[^ ^257^ ^]^ Moreover, nanowires with a large aspect ratio, textured in a specific crystal plane direction, can be mass‐produced. We previously reported an analysis of the electrical properties of Co nanowires with a minimum diameter of 16 nm fabricated by a bottom‐up fill method using porous nano‐templates.^[^ ^53^ ^]^ These electrodeposited Co nanowires demonstrated high crystallinity, and the textured plane direction changed from (002) to (100) with decreasing diameter (Figure 13c). The resistivity of a single Co nanowire measured by the in‐situ four‐probe method slowly increased from 9.4 to 15.3 µΩ cm as the diameter was reduced from 130 to 30 nm. However, the resistivity increased dramatically to 39.7 µΩ cm when the diameter decreased to 16 nm. The increase in grain scattering due to the decreasing grain size, the change in the EMFP value, and the switching of the main plane direction presumably played a significant role. Compared with the thin‐film resistivity, Co nanowires exhibited a relatively high resistivity value due to the increased surface scattering caused by the dimension difference and confining effect. However, the Co nanowire prepared by ED exhibited a lower resistivity value than that of a similar 1D shape.

Like the conventional Cu dual‐damascene process, bottom‐up Co super‐filling is achieved through a combination of additives, such as accelerators, suppressors, and levelers.^[^ ^258^, ^259^ ^]^ Because the Co‐precipitation and proton reduction in aqueous solutions occur at similar overpotentials, the control of hydrogen evolution is an essential factor for void‐free super‐conformal Co filling. Studies on suppressing hydrogen evolution, a competitive reaction, are underway by adding additives like boric acid, benzimidazole, and 2‐mercaptobenzimidazole.^[^ ^260^, ^261^, ^262^ ^]^ For example, boric acid adsorbs onto active surface sites and blocks the reduction of protons, thereby increasing the current deposition efficiency and affecting suppressor adsorption via pH buffering.^[^ ^260^ ^]^ Moreover, boric acid adsorbed on the electrode surface forms a complex with the Co ions in the electrolyte to prevent cobalt hydroxide precipitation, enabling subsequent Co nucleation and uniform growth. In addition, the pH gradient within the features plays a vital role in the super‐conformal deposition. When the externally applied current is lower than the diffusion‐limiting current for H^+^ reduction, the pH increases through H^+^ consumption at the base of the feature. The relatively elevated pH at the base thermodynamically promotes the reduction of CoOH^+^, an intermediate in the Co‐plating reaction, to enable super‐filling (Figure 13d).^[^ ^263^ ^]^

A few aspects must be improved in Co interconnects to prepare for future technology node interconnect scaling. Although the high cohesive energy of Co allows the reduction of barrier thickness, the TiN barrier for the Co line still exhibited a high resistivity. Moreover, the step coverage or wettability for ED became important for the MOL process, in which the CVD method was mainly used, as the material changed from W to Co.^[^ ^60^, ^264^ ^]^ These issues can be addressed by introducing Co‐based alloys. Metal‐based single‐layer alloys can replace the barrier/liner structure and improve the barrier property by forming an amorphous structure or using the GB stuffing effect.^[^ ^265^, ^266^ ^]^ CoTa, CoW, and CoMo have been examined as suitable monolayer materials to replace the conventional Cu damascene‐based TaN/Ta layer.^[^ ^51^, ^267^, ^268^ ^]^ The 3‐nm CoTi_x_ demonstrated excellent adhesion and interdiffusion barrier layer properties on Co/CoTi_x_/SiO_2_/p‐Si.^[^ ^269^ ^]^ Amorphous CoTi_x_ can maintain a stable state up to 500°C, and Ti is gradually separated and dissociates SiO_2_. The dissociated Si and O react until all Ti atoms are consumed and inhibit the Co diffusion by constructing a Si‐O‐Ti structure (Figure 13e). The relative mobility of atoms in these alloys has inspired the design of novel materials that simultaneously exhibit low resistivity and thermal stability.

#### 3.2.2 Ruthenium

**Figure 14:**
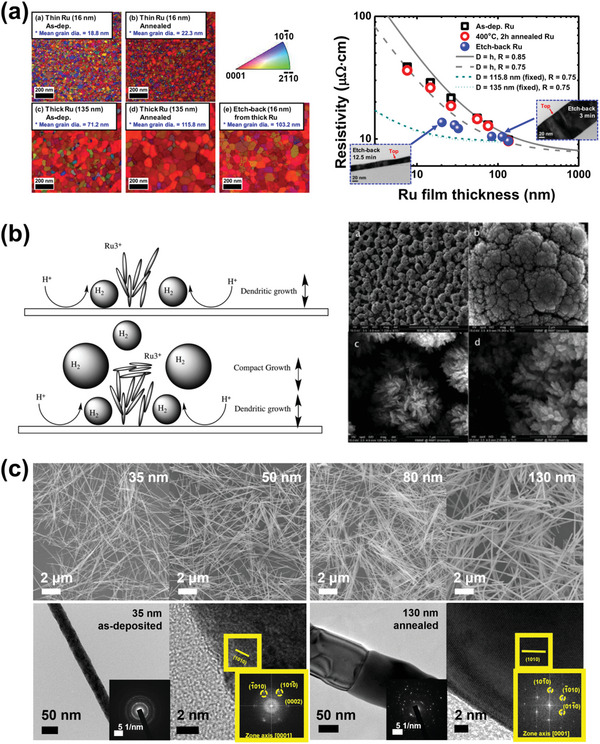
a) Automatic crystal orientation and phase mapping (ASTAR) of Ru thin film produced by the etch‐back process (left) and change in resistivity according to Ru thin film thickness (right). Reproduced with permission.^[^ ^57^ ^]^ Copyright 2018, Institute of Electrical and Electronics Engineers. b) Graphical illustration of Ru growth on the electrode where protons are reduced (left) and the various morphologies of electrodeposited Ru films (right). Reproduced with permission.^[^ ^274^ ^]^ Copyright 2014, The Electrochemical Society. c) Ru nanowires of various diameters electrodeposited by a bottom‐up growth process within a nanopore template (top). Ru fabricated by electrodeposition has an hcp structure, and the high‐resolution TEM image of Ru annealed at 600 °C for 1 h shows a highly ordered Ru microstructure (bottom). Reproduced with permission.^[^ ^54^ ^]^ Copyright 2022, Elsevier.

Ru is one of the most studied metals as a next‐generation wiring material. As shown in Figure 11, the FOM value of Ru is 3.81 × 10^−16^ Ω m^2^, which is 1.76 and 1.27 times lower than those of Cu and Co, respectively. Ru has become one of the most promising interconnect materials beyond 7‐nm node technology because of its barrierless process prospective and superior EM reliability owing to its high cohesive energy.^[^ ^270^ ^]^ Compared to the Cu control, the Ru showed almost immortal EM performance and higher TDDB life expectancy at ultra‐low *k* (*k* = 2.4).^[^ ^59^ ^]^ Early experimental results indicate that Ru has a resistivity of 16.5–15.7 µΩ cm in the range of 900–1250 cm^2^ in a dual‐damascene structure filled by a CVD method, which is three times greater than that of Cu with the same structure.^[^ ^270^ ^]^ However, the electron scattering of Ru can be reduced by optimizing the process. Ru deposited by ALD or a Ru line with an additional adhesion layer has a resistivity of 17–12 µΩ cm in 200–300 cm^2^.^[^ ^59^ ^]^ Ru wire prepared by a thin‐film deposition–annealing–etch‐back process shows a 30% reduction in resistivity at 68 nm^2^ compared to the conventional damascene Ru wire (**Figure** **14**a).^[^ ^57^ ^]^ Ru that underwent conformal metal deposition and anisotropic metal etch processing has a 27 µΩ cm resistivity at 58 nm^2^.^[^ ^271^ ^]^ Such Ru is expected to be crossover with Cu below a 17‐nm CD and is becoming a promising next‐generation substitute with outstanding electrical properties.

There have been few reports on utilizing the ED method for Ru. In addition to interconnections in semiconductors, many studies have been conducted on Ru to replace other noble metals, such as Au and Pt, in energy storage and catalysis. However, electrochemical development remains insufficient because of its industrial importance.^[^ ^272^, ^273^ ^]^ Due to the slow ED kinetics and competitive hydrogen evolution, the inability to easily control the morphology complicates Ru ED (Figure 14b).^[^ ^274^ ^]^ Obtaining pure metallic Ru films is challenging even if Ru is electrodeposited because it tends to be oxidized in aqueous solutions.^[^ ^275^ ^]^ Moreover, when reduced around the cathode, Ru acts as a catalyst by decomposing water and becoming a hydrogen‐generating spot; therefore, producing Ru in the desired shape is difficult.^[^ ^273^, ^276^ ^]^ We recently synthesized Ru nanowires by adding a chloride‐based additive and a buffer to suppress hydrogen generation around the electrode (Figure 14c). Different Ru‐Cl complexes can be formed with the RuCl_3_ precursor in an aqueous solution as the concentration of chloride ions changes. Because the complexes have different reduction potentials depending on the number and type of ligands, they are reduced at a specific voltage. Ru nanowires with high aspect ratios were synthesized using the designed plating solution on a nanoscale‐confined porous template.^[^ ^54^ ^]^ The resistivity increase rate in terms of the diameter and electron scattering parameters of the electrodeposited hcp Ru nanowires were derived. After the inner walls of the nanopores were coated with SiO_2_ using the ALD method, the bottom‐up‐filled Ru nanowires did not show interdiffusion even after annealing at 450 °C for 1 h, indicating their thermal stability. Currently, studies are being conducted to fill trenches using the ALD method or to provide Ru wiring using the etching method. Nevertheless, the ALD method has similar drawbacks as CVD and requires a long process.

**Figure 15:**
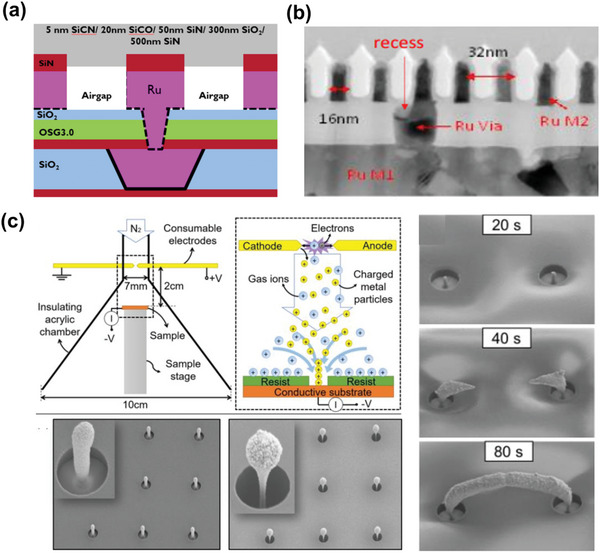
a) IMEC proposed the Ru semi‐damascene scheme. The convenient selective etching of Ru allows the fabrication of air‐gaps with a low dielectric constant. Reproduced with permission.^[^ ^277^ ^]^ Copyright 2021, Institute of Electrical and Electronics Engineers. b) Cross‐sectional TEM images of Ru semi‐damascene and AGs reported by IMEC. Reproduced with permission.^[^ ^278^ ^]^ Copyright 2020, Institute of Electrical and Electronics Engineers. c) Self‐aligned Ru interconnects fabricated by vapor electrodeposition. Introducing the new process can directly adjust the size of the enclosed AG and the diameter of the interconnect. Reproduced with permission.^[^ ^283^ ^]^ Copyright 2020, Institute of Electrical and Electronics Engineers.

In addition to the size effect of Ru, its excellent EM resistance enables the introduction of new processes. The industry has been pursuing the design technology co‐optimization (DTCO) of chip designs to address the increasing technology cost and complexity while simultaneously providing the historically expected improvements. However, the industry considers the system technology co‐optimization (STCO) strategy as the complexity increases. One crucial process in STCO is the introduction of semi‐damascene integration to solve the *RC* trade‐off problem. Metallization progresses from the existing dual‐damascene process to a hybrid process and then semi‐damascene using an air‐gap (AG) dielectric. This includes the direct etching (subtractive metallization) of the metal to achieve higher aspect ratio lines and partial or whole AG integration to cope with the capacitance increase (**Figure** **15**a,b).^[^ ^277^, ^278^ ^]^ Ru allows for lower process complexity and costs related to barrierless integration and larger grain size owing to the convenience of selective etching compared to Cu.^[^ ^279^ ^]^ In addition, in contrast to the Cu AG method, which requires encapsulation of the hermetic dielectric layer to prevent oxidation, Ru has the advantage of limited surface oxidation.^[^ ^280^ ^]^ Barrierless Ru combined with semi‐damascene allows for a smaller metal pitch.^[^ ^62^, ^278^, ^281^ ^]^ Moreover, an adequate adhesion‐promoting layer to Ru‐dielectric interfaces provides mechanical integrity equivalent to the existing dual‐damascene Cu BEOL, even for a shape with an aspect ratio of 3.^[^ ^282^ ^]^ Self‐aligning Ru interconnects technology with a gas‐phase ED process performed with an adjustable AG has been developed (Figure 15c).^[^ ^283^ ^]^

#### 3.2.3 Binary Intermetallic Compounds

**Figure 16:**
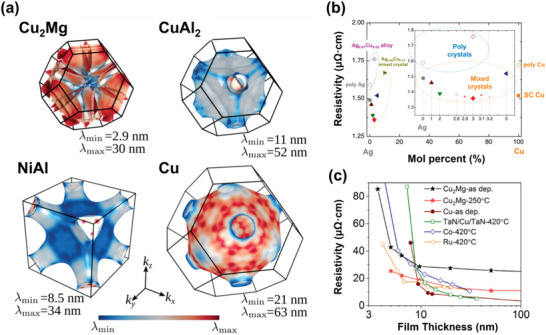
a) Calculated Fermi surfaces of the intermetallic compounds and Cu. The surface colors represent the anisotropic k‐dependent EMFP. Reproduced with permission.^[^ ^284^ ^]^ Copyright 2021, American Institute of Physics publishing. b) Abnormal resistivity drop in Ag–Cu alloys of specific composition. An alloy with a well‐ordered crystal structure may have a lower resistivity than a pure metal. Reproduced with permission.^[^ ^293^ ^]^ Copyright 2014, Nature publishing group. c) Comparison of the thickness‐dependent resistivities of Cu_2_Mg, Cu, Co, and Ru. Cu_2_Mg annealed at 250 °C for 30 min shows low resistivity scaling. Reproduced with permission.^[^ ^295^ ^]^ Copyright 2021, Elsevier.

In addition to alternative elemental metals such as Co or Ru, intermetallic compounds have evolved as candidates (**Figure** **16**a).^[^ ^241^, ^284^ ^]^ Although binary and ternary systems have been extensively studied in the metallurgy field, few reports have been on the resistivities in the 10‐nm band. According to Nordheim's rule, the residual resistivity of binary alloys exhibits a parabolic curve proportional to the mole fraction of the two pure metals.^[^ ^285^ ^]^ However, a sharp drop in resistivity occurs at stoichiometric concentrations associated with forming long‐range chemical ordering.^[^ ^53^ ^]^ For instance, CuAu (4.0 µΩ cm), Cu_3_Au (4.2 µΩ cm), Cu_2_Mg (4.5–7.1 µΩ cm), Cu_3_Ge (6.0 µΩ cm), CuAl_2_ (7.6–8.0 µΩ cm), and NiAl (8.0–10.0 µΩ cm) have resistivity values lower than 10.0 µΩ cm at room temperature.^[^ ^286^, ^287^, ^288^, ^289^, ^290^, ^291^, ^292^ ^]^ One study reported that adding a small amount of Cu impurity to Ag resulted in a lower resistivity than pure Ag because of the inhibitory effect of the electronic structure and vacancy formation (Figure 16b).^[^ ^293^ ^]^

In addition to their prominent resistive properties, intermetallic compounds exhibit strong chemical bonding between neighboring atoms, which usually serves as an advantage in diffusion‐related reliability. As mentioned at the end of Section 3.2.1, the relative mobility of metals within the alloy may be helpful in future wiring formation.^[^ ^269^ ^]^ In particular, because compounds containing Mg, Al, and Ti have higher oxide formation energies than that of SiO_2_, they can react with SiO_2_ and adequately adhere to dielectric insulators.^[^ ^294^ ^]^ Cu_2_Mg has a better size effect in resistivity with physical scaling down than Cu and Co, in addition to a low resistance value similar to Ru (Figure 16c).^[^ ^295^ ^]^ Nevertheless, when the trench is filled with Cu_2_Mg by sputtering, surface diffusion becomes difficult because the activation energy of Mg is higher than that of Cu. Thus the composition of Mg is lowered, and the stoichiometry cannot be satisfied. When annealed for 30 min, a thick MgO layer is formed, leading to fatal dielectric current leakage. Therefore, intermetallic compounds containing Al have come forth.^[^ ^296^, ^297^ ^]^ When a compound containing Mg and Al is annealed, an interfacial layer (MgO or Al_2_O_3_) is created at the interface of the compound and SiO_2_. However, the difference in the oxide growth rates, caused by the diffusivity difference between cations and anions inside the two materials, leads to the formation of a thinner Al_2_O_3_, which has a slower oxide growth rate.^[^ ^284^ ^]^

NiAl and CuAl_2_ have been studied beyond 3‐nm node metallization materials.^[^ ^298^ ^]^ The strong chemical bonding (or enormous cohesive energy) between Cu–Al or Ni–Al leads to a slow reaction between SiO_2_ and Al. The self‐limiting thickness of Al oxide indicates the possibility of liner‐ and barrier‐free interconnects.^[^ ^299^ ^]^ However, for NiAl, when the thin film thickness is reduced to 15 nm or less, there are some disadvantages, such as the high resistivity rate due to the non‐stoichiometric surface oxide layer and poor gap‐filling property.^[^ ^296^, ^298^ ^]^ CuAl_2_ shows good gap‐filling and lower resistivity than the TaN/Cu/TaN structure below 8 nm.^[^ ^298^ ^]^ The introduction of the ALD process is expected to accelerate the introduction of intermetallic compounds. Properly controlled process conditions can conformally fill the intermetallic compound into the trench structure by adjusting the thickness of the sub‐nanometer.^[^ ^300^ ^]^ Intermetallic compounds are attractive substitutes for resistivity and material diffusion; however, many challenges remain. Therefore, further studies should be conducted in the future.

#### 3.2.4 MAX Phases

**Figure 17:**
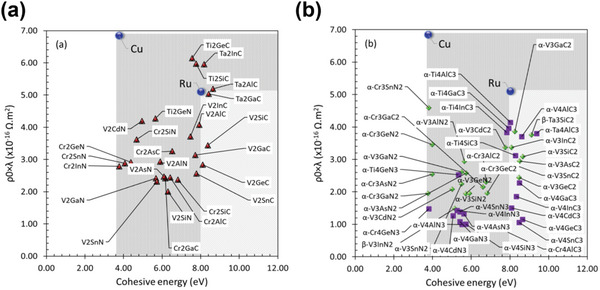
FOM for stable MAX phases. The a) 211 stoichiometry and the b) 312 and 413 stoichiometries are expected to have favorable properties concerning Cu and Ru. Reproduced with permission.^[^ ^46^ ^]^ Copyright 2021, American Physical Society.

MAX or M*_n_*_+1_AX*_n_* (M: early transition metal; A: A‐group element located in columns 13 or 14 of the periodic table; X: either C or N; and n: 1,2, or 3) phases are layered, hexagonal, and early transition metal carbides and nitrides that have garnered significant attention owing to their unique combination of both metallic and ceramic properties, such as high melting points, large electrical conductivity, and excellent oxidation resistance.^[^ ^301^, ^302^, ^303^, ^304^ ^]^ A myriad of combinations is possible with multiple candidate elements for each stoichiometry (*n* = 1–3) and polymorphs due to the change in the order of the layered structures.^[^ ^305^, ^306^, ^307^ ^]^ The intrinsic layered structure is less sensitive to the structure or grain size, making it suitable for scaled interconnect applications. IMEC reported a first‐principles simulation based on DFT calculations for ≈170 different MAX compound candidates by benchmarking the methodology conducted on existing alternative materials.^[^ ^46^ ^]^ The two properties, *ρ*_0_  × *λ* for resistivity scaling potential and cohesive energy for reliability are the essential requirements for interconnect materials to act as determinants for candidate material selection. Among the 170 MAX compounds investigated, 69 stable states showed superior characteristics to Cu for both proxies, and 24 were superior to Ru (**Figure** **17**). However, many new substances have not yet been studied due to the various possible combinations. Nevertheless, additional studies should be conducted to materialize MAX compounds for actual wiring. A precursor for synthesizing the intended layered structure must be developed and deposited within a narrow line in a hexagonal (001) textured form because of the large anisotropy of electrical conductivity. Furthermore, the high process temperature for MAX phase synthesis should be optimized to be produced within a relatively low BEOL thermal budget.

## 4 Summary and Outlook

New materials are needed to enable the continuous expansion of interconnect technology. This article provides a multifaceted and comprehensive review and summary of the challenges that BEOL has faced to keep up with the rapidly shrinking FEOL area. The reduction of the interconnect structure to the nanoscale causes three significant problems. First, the reduced trench width increases electron scattering within the metal conductor, dramatically increasing the line resistance. Second, the increased electron scattering generates high heat in the device, which accelerates the movement of atoms and the malfunction of the device. Third, the additional layers required to increase the reliability of the interconnect occupy the space for the metal conductor, which leads to increased resistance and makes the process difficult.

Therefore, next‐generation barrier materials that reduce the thickness of the barrier and liner to sustain Cu wiring scaling have been proposed. Ru‐based alloys are barrier materials that can replace the existing TaN/Ta material because of their excellent diffusion prevention properties and easy processing. Essentially, 2D materials at the sub‐nano scale can dramatically reduce the barrier/liner thickness. However, some of the remaining problems that must be solved include processing and high vertical resistance. SAM is a molecular‐sized barrier that can provide various functions by replacing the head and terminal groups. However, the relatively high temperature of the BEOL process is an obstacle to using SAM. Although HEAs have excellent anti‐diffusion properties owing to their cocktail effect, more research is needed to apply them to the process.

Finally, we are currently exploring promising next‐generation barrierless conductor materials. Only a few candidates have been selected based on their size effect on resistivity and reliability. Co‐filling based on ED is friendly to the dual‐damascene process and successfully replaces the conventional W contact and lowermost layer of the Cu interconnect. There are ongoing discussions on filling Co into smaller trench structures. Co still requires a relatively high‐resistivity barrier, although it has a thinner barrier than Cu wiring. Therefore, Ru has become a prominent next‐generation material that surpasses Co. The low resistivity size effect and high cohesiveness of Ru make it the most studied material because it eliminates the need for a barrier within a smaller width. In addition, in contrast to conventional Cu, easy selective etching enables semi‐damascene to convert low‐k dielectrics into air‐gaps, which can dramatically lower the RC delay of the interconnect. Many researchers are exploring new material concepts in preparation for the generation beyond Ru. For example, some intermetallic compounds exhibit lower resistivity scaling than that of Co and Ru. In addition, the relative movement of atoms during heat treatment forms a natural oxide layer between low‐k interfaces, enabling barrierless wiring. Finally, several newly proposed layered and hexagonal MAX phases exhibit superior properties to Ru in terms of their material properties. In the sub‐nm technology node, the MAX phase may be hopeful.

The results from various academic and industrial circles are discussed and reviewed in this review. However, there are still many challenges to the practical application of each material in the industry. Currently, close connection and cooperation between academia and industry must begin to solve the problems facing the semiconductor metallization process and meet the demands of the IC generation.

## Conflict of Interest

The authors declare no conflict of interest.

## Author Contributions

J.H.M.: Conceptualization, Investigation, Writing—Original draft preparation, Visualization; E.J.: Investigation, Writing—Editing; S.K.: Investigation, Visualization; T.K.: Investigation; E.O.: Visualization; K.L.: Investigation; H.H.: Investigation; Y.K.K.: Writing—Review and editing, Supervision, Funding acquisition
